# Systems genetics analysis reveals immune mediators of coronavirus disease severity in mice

**DOI:** 10.1093/genetics/iyag209

**Published:** 2026-08-21

**Authors:** Ellen L Risemberg, Sarah R Leist, Alexandra Schäfer, Kalika D Kamat, Timothy A Bell, Pablo Hock, Kara L Jensen, Colton L Linnertz, Darla R Miller, Ginger D Shaw, Fernando Pardo Manuel de Villena, Ralph S Baric, Martin T Ferris, William Valdar

**Affiliations:** Department of Genetics, University of North Carolina at Chapel Hill, Chapel Hill, NC 27517, United States; Curriculum in Bioinformatics and Computational Biology, University of North Carolina at Chapel Hill, Chapel Hill, NC 27517, United States; Department of Epidemiology, University of North Carolina at Chapel Hill, Chapel Hill, NC 27517, United States; Department of Epidemiology, University of North Carolina at Chapel Hill, Chapel Hill, NC 27517, United States; Department of Genetics, University of North Carolina at Chapel Hill, Chapel Hill, NC 27517, United States; Curriculum in Bioinformatics and Computational Biology, University of North Carolina at Chapel Hill, Chapel Hill, NC 27517, United States; Department of Genetics, University of North Carolina at Chapel Hill, Chapel Hill, NC 27517, United States; Department of Genetics, University of North Carolina at Chapel Hill, Chapel Hill, NC 27517, United States; Department of Epidemiology, University of North Carolina at Chapel Hill, Chapel Hill, NC 27517, United States; Department of Genetics, University of North Carolina at Chapel Hill, Chapel Hill, NC 27517, United States; Department of Genetics, University of North Carolina at Chapel Hill, Chapel Hill, NC 27517, United States; Department of Genetics, University of North Carolina at Chapel Hill, Chapel Hill, NC 27517, United States; Lineberger Comprehensive Cancer Center, University of North Carolina at Chapel Hill, Chapel Hill, NC 27517, United States; Department of Genetics, University of North Carolina at Chapel Hill, Chapel Hill, NC 27517, United States; Lineberger Comprehensive Cancer Center, University of North Carolina at Chapel Hill, Chapel Hill, NC 27517, United States; Department of Epidemiology, University of North Carolina at Chapel Hill, Chapel Hill, NC 27517, United States; Lineberger Comprehensive Cancer Center, University of North Carolina at Chapel Hill, Chapel Hill, NC 27517, United States; Department of Microbiology and Immunology, University of North Carolina at Chapel Hill, Chapel Hill, NC 27517, United States; Department of Genetics, University of North Carolina at Chapel Hill, Chapel Hill, NC 27517, United States; Department of Genetics, University of North Carolina at Chapel Hill, Chapel Hill, NC 27517, United States; Lineberger Comprehensive Cancer Center, University of North Carolina at Chapel Hill, Chapel Hill, NC 27517, United States

**Keywords:** collaborative cross, coronavirus, SARS-CoV, SARS-CoV-2, immune system, immunology, Bayesian statistics, missing data imputation, variable selection, QTL mapping, mediation analysis, genotype-by-treament, GxT

## Abstract

Severe disease following infection with SARS-CoV or SARS-CoV-2 is driven in part by genetically regulated immune responses that promote lung injury. To study genetic mechanisms of immune-mediated coronavirus pathogenesis, we screened the Collaborative Cross (CC) panel for *Mus musculus* strains that are phenotypically divergent with respect to coronavirus susceptibility. We identified CC006/TauUnc and CC044/UncJ as susceptible and resistant, respectively, and crossed them in an intercross to produce a genetic mapping population. Previously, we mapped genetic loci associated with disease severity, including *HrS43*, which is both conserved across viruses and between mouse and human. Here, we incorporate immune cell data from flow cytometry and perform a systems genetics analysis to resolve immune pathways linking host genetic loci to disease outcomes. We identify: (1) immune predictors of disease severity, as revealed by Bayesian variable selection; (2) extensive genetic regulation of the immune system at homeostasis and in response to infection, as revealed by infection-stratified and combined genotype-by-treatment (GxT) QTL mapping; and (3) candidate causal pathways linking genetic loci, immune responses, and disease severity, as revealed by context-dependent Bayesian mediation analysis. Our analysis reveals distinct patterns of virus specificity across biological layers: immune effects on disease severity appear largely consistent across viruses; genetic architecture is both shared and virus-specific; and mediating immune traits are in most cases virus-dependent. Notably, *HrS43* appears to influence disease severity through distinct immune mediators in SARS-CoV versus SARS-CoV-2, demonstrating that conserved genetic susceptibility can drive virus-specific immunopathology with translational relevance across species.

## Teaser

People vary widely in how sick they become after coronavirus infection, partly because of inherited differences in immune responses. In this study, Risemberg et al. used mice to identify genetic factors and immune cells influencing susceptibility to SARS-CoV and SARS-CoV-2. Many genetic effects on the immune system were consistent across the two viruses, but the immune pathways leading to severe disease often differed. In particular, *HrS43*, a genetic region linked to coronavirus disease severity in both mice and humans, acted through different immune cells depending on the virus. These results improve understanding of how genetic variation shapes immune responses to coronavirus infection.

## Introduction

Zoonotic coronaviruses, including SARS-CoV, MERS-CoV, and SARS-CoV-2, have caused major epidemics with highly variable clinical outcomes (Zhong et al. 2003; Hijawi et al. 2013; Zhou F et al. 2020; Zhou P et al. 2020). Coronaviruses circulating in animal reservoirs remain poised for human emergence (Menachery et al. 2015) and expanding human-animal contact is expected to increase the risk of zoonotic transmission (Carlson et al. 2022), highlighting the importance of understanding conserved host determinants of disease susceptibility. Despite a broad and sustained effort to understand biological mechanisms of SARS-CoV-2 pathogenesis, many factors leading to severe coronavirus disease remain incompletely understood.

Individual responses to viral infection range from asymptomatic to lethal and are affected by a complex interplay between host, viral, and environmental factors, with host genetics playing a critical role. Host genetics have been shown to regulate response to infection in the human population across a range of viruses, such as influenza A (Clohisey and Baillie 2019; Gounder and Boon 2019), hepatitis C (O’Brien et al. 2019), and herpes simplex encephalitis (Zhang SY 2020). Naturally, many host genes associated with infectious disease susceptibility are immune genes themselves (Zhang SY et al. 2007; Thomas et al. 2009; The GenISIS Investigators 2012) or are noncanonical immune genes thought to act through immune-related mechanisms (Patin et al. 2012; Sologuren et al. 2018; Zhang SY et al. 2018).

Given the heterogeneity in response to coronavirus infection, which was seen in SARS and MERS (Yin and Wunderink 2018) and is especially pronounced in COVID-19, the disease caused by SARS-CoV-2 (Huang et al. 2020), genetics is likely to play a role in regulating coronavirus disease severity. Indeed, heritability of SARS-CoV-2 susceptibility has been estimated to be between 33 and 70% (Brown et al. 2024), and a combination of candidate gene studies (for SARS and MERS) and genome-wide association studies (GWAS; for COVID-19) have identified several genes and loci associated with coronavirus infection and disease severity (Lin et al. 2003; Itoyama et al. 2004; He et al. 2006; LoPresti et al. 2020; The Severe Covid-19 GWAS Group 2020; COVID-19 Host Genetics Initiative 2021; The GenOMICC Investigators 2021; Baek et al. 2022; Horowitz et al. 2022; Kousathanas et al. 2022; Pairo-Castineira et al. 2023).

Like other severe viral illnesses (Damjanovic et al. 2012; Bergeron and Tripp 2021), severe coronavirus disease involves substantial immune-mediated pathology (Cameron et al. 2008; Cao 2020), and several immune traits have been implicated in severe SARS and COVID-19. Increased infiltration of inflammatory macrophages and neutrophils into the lung and airway (Chua et al. 2020; Liao et al. 2020; Dorward et al. 2021; Grant et al. 2021; Melms et al. 2021; Chen et al. 2022), decreased expression of MHC class I and II genes (Giamarellos-Bourboulis et al. 2020; Schulte-Schrepping et al. 2020; Silvin et al. 2020; Asaba et al. 2025), and decreased peripheral T cells along with increased T cell activation (Giamarellos-Bourboulis et al. 2020; Kuri-Cervantes et al. 2020; Laing et al. 2020; Lucas et al. 2020; Song et al. 2020) are hallmarks of severe COVID-19. Increased lung macrophages (Nicholls et al. 2003) and decreased peripheral T cells (Li T et al. 2004) have also been associated with severe SARS.

Together, these observations that both host genetics and inter-individual variation in immune responses contribute to disease severity suggest that genetic variants associated with disease severity are likely to exert their effects by modulating key immune pathways. GWAS, particularly when integrated with transcriptomic and cellular endophenotypes, have implicated immune pathways in severe COVID-19, including cytokine signaling and mobilization of inflammatory cells (The GenOMICC Investigators 2021; Kousathanas et al. 2022; Pairo-Castineira et al. 2023). Complementary studies on cells derived from susceptible patients have further implicated genetically driven dysregulation in innate immune signaling (Van Der Made et al. 2020; Zhang Q et al. 2020). Notably, genetic studies implicating immune pathways have also informed therapeutic development: a GWAS on severe COVID-19 (The GenOMICC Investigators 2021) identified a therapeutic gene target in a cytokine signaling-related gene, *TYK2*, that resulted in an effective treatment for severe COVID-19 (RECOVERY Collaborative Group 2022).

Given accumulating evidence from human genetic studies that immune pathways mediate genetic effects on coronavirus disease severity, complementary experimental systems are valuable for evaluating causal relationships under controlled conditions. Mouse models of infection provide the ability to study genetically driven variation in immune responses while controlling key sources of variation that confound human studies, including viral strain and dose, timing of infection, and prior immune exposure. In addition, mice enable consistent and comprehensive immune phenotyping across tissues and disease stages, including access to lung tissue early in infection, which is rarely feasible in human cohorts.

The utility of mice for studying host genetic susceptibility to coronavirus infection was first demonstrated in studies of mouse hepatitis virus (MHV) in laboratory strains (Ohtsuka and Taguchi 1997). Subsequent development of mouse-adapted SARS-CoV (SARS-CoV MA15; Roberts et al. 2007) and SARS-CoV-2 (SARS-CoV-2 MA10; Leist et al. 2020), which recapitulate many phenotypic features of human disease, has facilitated the study of these viruses in mouse models. Classical inbred mouse strains have been used to study many aspects of coronavirus disease, including determinants of severe SARS-CoV disease (Gralinski et al. 2013) and pan-coronavirus vaccine efficacy (Adams et al. 2023).

The emergence of genetically diverse mouse reference panels, such as the Collaborative Cross (CC), has expanded the scope of these analyses by capturing levels of genetic and phenotypic diversity comparable to that in human populations, while retaining the advantages of a replicable inbred system (Collaborative Cross Consortium 2012). The CC has been effectively deployed to study genetics of immune homeostasis (Phillippi et al. 2014), susceptibility to immune-mediated diseases such as food allergy (Orgel et al. 2019; Risemberg et al. 2024) and inflammatory bowel disease (Rogala et al. 2014), and susceptibility to a number of pathogen infections, including influenza A (Ferris et al. 2013; Maurizio et al. 2018; Noll et al. 2020), Ebola (Rasmussen et al. 2014; Schäfer, Marzi et al. 2024), Crimean-Congo hemorrhagic fever (Rao et al. 2025), tuberculosis (Smith et al. 2022), Salmonella (Scoggin et al. 2022), and West Nile virus (Green et al. 2017). Importantly, these improved host and viral models have facilitated the identification of several host susceptibility loci for coronavirus disease (Gralinski et al. 2015, 2017; Schäfer et al. 2022; Leist et al. 2024; Schäfer, Gralinski et al. 2024).

Previously, we demonstrated that some host susceptibility loci are conserved across coronaviruses in mice, and are shared between mouse and human (Schäfer et al. 2022; Leist et al. 2024; Schäfer, Gralinski et al. 2024). These findings suggest that genetic determinants of coronavirus disease severity may reflect conserved biological pathways relevant both to human infection and to the risk posed by future emergent coronaviruses, in addition to the continued global circulation of SARS-CoV-2. Here, we apply a systems genetics approach, integrating immune phenotyping with genetic and disease severity data, to investigate immune-mediated mechanisms of genetic susceptibility. We present a statistical framework for jointly modeling correlated immune traits across infection conditions and linking host loci to disease outcomes. Using this framework, we identify both virus-specific and shared immune pathways through which conserved genetic susceptibility may shape coronavirus immunopathology.

## Materials and methods

### Experimental methods

#### Mouse acquisition, production, and usage

Mouse studies were performed in strict accordance with the recommendations in the Guide for the Care and Use of Laboratory Animals of the National Institutes of Health. All mouse studies at UNC (Animal Welfare Assurance #A3410-01) were performed using protocols approved by the UNC Institutional Animal Care and Use Committee (IACUC) in a manner designed to minimize pain and suffering in infected animals.

Collaborative Cross mice were purchased from the Systems Genetics Core Facility at UNC Chapel Hill between 2/2019 and 11/2020. We generated and maintained sublines of CC006 and CC044 that had identical MHC loci, defined as both having C57BL/6J haplotypes between 34 and 35.5 Mb (b38) on chromosome 17. From these mice, we generated an ${\text{F}}_{2}$ mapping population as previously described (Leist et al. 2024). Briefly, we crossed CC006 and CC044 mice in both directions to generate reciprocal ${\text{F}}_{1}$s. We then crossed these ${\text{F}}_{1}$s in all 4 possible reciprocal matings to generate ${\text{F}}_{2}$ animals. In this way, potential maternal, mitochondrial, and sex chromosome effects are balanced across our population. At weaning, mice from different birth cages were co-housed together, to ensure that parental or birth-cage effects were distributed across experimental cages. All breeding was conducted in the Department of Comparative Medicine, SPF facilities at UNC Chapel Hill. Mice were given *ad libitum* food and water and kept on a 12:12 light:dark cycle. Experimental mice were transferred into an ABSL-3 laboratory at 9–15 wk of age, acclimated for one week and then infected. The ABSL3 was kept on a 12:12 light:dark cycle, and mice were given *ad libitum* access to food and water.

#### Viruses

The generation of recombinant mouse-adapted viruses—SARS-CoV MA15 (mouse-adapted SARS-CoV-Urbani_AY278741) and SARS-CoV-2 MA10 (mouse-adapted SARS-CoV-2_Wuhan_MN908947)—used in this study was previously described (Roberts et al. 2007; Becker et al. 2008; Leist et al. 2020). All experiments were performed in a certified ABSL-3 laboratory, and personnel wore appropriate personal protective gear.

#### Plaque assays

Stock viruses and replication competent viral particles in mouse lungs were quantified via plaque assay as reported previously (Roberts et al. 2007; Becker et al. 2008; Leist et al. 2020). Briefly, lung tissue (caudal lobe) was homogenized, and monolayers of Vero E6 cells (ATCC CCL-81) were infected with 10-fold dilutions of supernatant. After incubation for 1 h at 37 ^∘^C, infected monolayers were overlayed with agarose in cell media. Resulting plaques were counted after 48 h (SARS-CoV) or 72 h (SARS-CoV-2) at 37 ^∘^C and 5% CO${\text{CO}}_{2}$ and final amounts were calculated.

#### Infection

Mice were lightly anesthetized via isoflurane inhalation and infected intranasally with $10^{4}$ plaque-forming units (PFU) of SARS-CoV MA15 and SARS-CoV-2 MA10 in a total of 50 μ L PBS or PBS only as mock infection-control. Following infection, mice were assessed daily for weight loss and monitored for mortality. At 4 d post infection (dpi), mice were euthanized via isoflurane overdose followed by bilateral thoracotomy. Mice were perfused with sterile 1xPBS prior to tissue collection. From each animal, the inferior lobe of the lung was collected for histological analysis, from which a congestion score was determined, and the caudal lobe for measurement of lung titer. Samples were stored at $-80\circ^{C}$. The entire left lobe was used immediately for the isolation of single cell suspension for flow analysis.

#### Flow cytometry staining

We derived three panels of flow cytometry markers to identify granulocyte, myeloid, and lymphoid populations (panel details in Supplementary Table 1). Perfused lungs were incubated in digestion buffer (RPMI 1640, 10% fetal bovine serum, 15 mM HEPES, 2.5 mg/ml collagenase A (Worthington), 1.7 mg/ml DNase I (Sigma)) for 2 h at 37 ^∘^C on a shaker. Cells were then processed through a 70-μm filter, followed by red blood cell lysis with ACK lysis buffer. Single cell suspensions were then diluted to a concentration of approximately $1\times 10^{7}$ cells/mL (approx. $1\times 10^{6}$ cells/100 μL). Samples were washed twice with wash buffer (1xPBS/1% FCS) and resuspended with 200 μL antibody mixture (1:200 dilution in staining buffer (1x HBSS, 1% FBS)) of the relevant staining panel (Supplementary Table 1) for 60 min at 37 ^∘^C. After staining, each sample was washed twice, and samples were fixed with 2% Formalin, stored in 1xPBS for 24 h at 4 ^∘^C, and then taken out of the BSL3 for analysis.

#### Flow cytometry data acquisition and analyses

Following staining, we ran samples on a LSRFortessa (Becton Dickinson), which is equipped with 5 lasers (355, 405, 488, 561, and 540 nm). Following acquisition, flow cytometry data were analyzed using FlowJo (v10.8.1) to identify a set of 105 immune cell traits, describing the proportional abundance of T cells, B cells, natural killer (NK) cells, macrophages, dendritic cells, and granulocytes (mast cells, basophils, eosinophils, and neutrophils), along with their functional activation states (CD25, CD69, PD1), antigen presenting function (MHC II), co-stimulatory molecule expression (CD80, CD86, CD40), trafficking receptor expression (CCR2, CCR7), and T cell differentiation state (effector memory, central memory, naïve). Single color compensation controls, fluorescence minus one (FMO) controls and experimental fcs files for lymphoid, myeloid and granulocyte panels were loaded into separate workspaces. A compensation matrix was generated and applied to each panel using the relevant controls. Each sample was quality controlled by removing any artifacts introduced through irregular sample flow or clumping. Single cells were then selected using forward and side scatter height and width. Finally, live cells were chosen by gating on cells excluded by the viability dye (LIVE/DEAD™Fixable Blue Dead Cell Stain Kit, Invitrogen). Each panel was then gated separately to generate lymphocyte, myeloid, and granulocyte traits followed by exporting frequencies of each cell type and mean fluorescence intensities of activation markers.

### Statistical methods

#### Data pre-processing

Immune traits were aggregated from three gated flow cytometry datasets, which were harmonized as described in Appendix A. All immune traits were then logit-transformed and random batch effects were removed (Appendix A). Within the subset of mice that were phenotyped for immune traits, some immune trait observations were missing at random due to technical issues with samples.

A statistic representing overall weight loss trajectory for each animal was calculated by measuring the area between a horizontal line at 100% and the line formed by the weight trajectory in terms of percentage of starting weight (illustrated in Supplementary Fig. 1a). This statistic was called area above the curve (AAC) and used throughout analyses as a primary measure of disease severity.

#### Variance partitioning and heritability estimation

Variance in immune traits explained by covariates and SNP-based heritability (Yang et al. 2017) were estimated by fitting the linear mixed model,


$$y_{i,j}=\mu +x_{\mathrm{sex},i}\beta_{\mathrm{sex}}+x_{\inf,i}\beta_{\inf}+b_{j}+u_{i}+\varepsilon_{i},$$

where $y_{i,j}$ is the phenotypic value for mouse *i* in batch *j*, *μ* is the intercept, $x_{\mathrm{sex},i}$ and $x_{\inf,i}$ are covariates for sex and infection status, respectively, and $\beta_{\mathrm{sex}}$ and $\beta_{\inf}$ are the corresponding fixed-effect coefficients, $b=(b_{1},\ldots ,b_{J})∼N(0,\sigma_{b}^{2}I)$ are random batch effects, $u=(u_{1},\ldots ,u_{n})∼N(0,\sigma_{g}^{2}G)$ are random polygenic effects based on the genomic relationship matrix G, and $\varepsilon =(\varepsilon_{1},\ldots ,\varepsilon_{n})∼N(0,\sigma^{2}I)$ are independent and normally distributed residual errors. G was estimated using the approach described by VanRaden (2008) and implemented in the AGHmatrix R package (Amadeu et al. 2023).

Immune traits for variance component analysis were processed as described in Data pre-processing but without batch effects removed, so that variance attributable to batch effects could be estimated and summarized. This model was fit with lme4qtl (Ziyatdinov et al. 2018), and variance components for random effects were extracted from the model using the VarCorr function and scaled by total phenotypic variance. Semi-partial $R^{2}$ were estimated for fixed effects using the approach described by Nakagawa and Schielzeth (2013) and implemented in the r2glmm R package (Jaeger et al. 2017).

#### Multicollinearity, PCA, and supervised classification

Multicollinearity was evaluated using multiple correlation coefficients (multiple $R^{2}$) and variance inflation factors (VIFs). Traits with VIFs exceeding 5, a common threshold for multicollinearity (James et al. 2013), were considered multicollinear. Principal components analysis (PCA) was performed on imputed data (using posterior means from the imputation model described below) with the R function prcomp. We then trained an elastic net multinomial regression classifier with glmnet (Friedman et al. 2010) to classify infection status from 105 immune traits. We tuned over mixing parameter α∈[0,1] and regularization strength $\lambda \in [10^{-3},1]$, performing five repeats of 10-fold cross-validation for each model. The best performing model, described in Results, had parameters α=0.1 and λ=0.112.

#### Bayesian imputation and variable selection

To determine which immune traits were most predictive of disease severity, we developed a Bayesian variable selection approach. A key complication was that immune trait measurements were missing for a subset of mice, with different traits missing for different individuals. Ignoring individuals with missing data would greatly reduce our sample size, and imputing data in a separate step prior to variable selection would underestimate uncertainty in the regression estimates and inclusion probabilities. We thus performed imputation and variable selection simultaneously within a custom Gibbs sampler, so that both sources of uncertainty were accounted for. In each iteration of the Gibbs sampler, missing immune data were imputed in an *imputation phase*, and effects were then estimated using the fully observed fixed-effect covariates and current imputed immune data in a *regression phase*. Full model details are given in Appendix B.

Briefly, in the *imputation phase*, missing immune data were imputed from a multivariate normal model conditioned on observed immune traits, sex, and infection status (Appendix B). In the *regression phase*, weight loss was modeled as a function of fixed-effect covariates and immune trait predictors subject to variable selection:


$$y_{i}=\mu +x_{\mathrm{sex},i}\beta_{\mathrm{sex}}+x_{\inf,i}\beta_{\inf}+\sum_{j}\delta_{j}z_{i,j}+\varepsilon_{i},$$

where $y_{i}$ is the weight loss (AAC) value for mouse *i*, *μ* is the intercept, $x_{\mathrm{sex},i}$ and $x_{\inf,i}$ are covariates for sex and infection status, respectively, and $\beta_{\mathrm{sex}}$ and $\beta_{\inf}$ are their corresponding fixed-effect coefficients, $z_{i,j}$ is the observed or imputed value of immune predictor *j* for mouse *i*, $\delta_{j}$ is the effect of predictor *j*, with δ under variable selection as described in the following paragraph and Appendix B, and $\varepsilon_{i}$ is the residual variance for mouse *i*.

Each of *q* immune traits contributed four predictors: a main effect, a trait-by-sex interaction, a trait-by-SARS-CoV interaction, and a trait-by-SARS-CoV-2 interaction, for a total of q×4 predictors under selection. Variable selection over these j∈{1,…,(q×4)} predictors was performed using Stochastic Search Variable Selection (SSVS) (George and McCulloch 1993, 1997), which assigns each predictor a “spike-and-slab” discrete normal mixture prior with a diffuse (“slab”) component and a tight (“spike”) component centered around 0. Specifically, each predictor *j* is assigned a binary inclusion indicator $\gamma_{j}\in \{0,1\}$, and its effect $\delta_{j}$ is drawn from the spike when $\gamma_{j}=0$ and from the slab when $\gamma_{j}=1$. The marginal posterior inclusion probability (PIP) for predictor *j* was estimated as the posterior mean of $\gamma_{j}$, i.e. the proportion of post-burn-in MCMC samples in which $\gamma_{j}=1$ (Appendix B). Sensitivity analyses suggested that results were robust to prior hyperparameter choices (Appendix B).

##### Posterior inclusion probability thresholds

A posterior inclusion probability (PIP) threshold of 0.5 is common when Bayesian variable selection is performed on orthogonal variables (Barbieri and Berger 2004), but is considered overly conservative with multicollinear predictor variables (Ghosh and Ghattas 2015). We therefore established a PIP threshold by permutation, permuting the outcome 1,000 times and running the sampler each time using a main-effects only model, thereby establishing a null distribution of PIPs when there is no association between immune traits and disease outcome. The 99th percentile of this null distribution, PIP = 0.23, was then used as our selection threshold. This threshold is conservative, since permutation diagnostics under the interaction model indicated even smaller null PIPs.

##### Effect size and direction

Posterior means of the virus-specific total effects for each immune trait were calculated as the sum of the posterior means of the main effect and the virus interaction effect, each conditional on inclusion in the model, i.e. for immune trait j∈[1,q] and virus v∈{1,2},


$$\begin{array}{rl} {\tilde{\delta }}_{{\mathrm{trait}}_{j},{\mathrm{virus}}_{v}} & =E[\delta_{{\mathrm{trait}}_{j}}|\gamma_{{\mathrm{trait}}_{j}}=1,y]\\ & \;+E[\delta_{{\mathrm{trait}}_{j}\times {\mathrm{virus}}_{v}}|\gamma_{{\mathrm{trait}}_{j}\times {\mathrm{virus}}_{v}}=1,y]. \end{array}$$

Sign concordance between SARS-CoV and SARS-CoV-2 was established for each immune trait by computing posterior draws of the virus-specific total effect (e.g. ${\tilde{\delta }}_{{\mathrm{trait}}_{j},{\mathrm{virus}}_{v}}=\delta_{{\mathrm{trait}}_{j}}+\delta_{{\mathrm{trait}}_{j}\times {\mathrm{virus}}_{v}}$) and calculating the posterior probability $P(\mathrm{sign}({\tilde{\delta }}_{{\mathrm{trait}}_{j},{\mathrm{virus}}_{1}})\;=$  $\mathrm{sign}({\tilde{\delta }}_{{\mathrm{trait}}_{j},{\mathrm{virus}}_{2}}))$. Traits with sign concordance in ≥80% of posterior samples were defined as having a consistent effect direction between SARS-CoV and SARS-CoV-2.

To compare the overall magnitude of sex and virus interaction effects, absolute posterior means of the sex interaction effects were compared with the average absolute posterior means of the SARS-CoV and SARS-CoV-2 interaction effects across predictive immune traits using a paired Wilcoxon signed-rank test.

##### Model performance and stability analysis

Mixing and convergence were evaluated via Gelman and Rubin’s $\hat{R}$ (Gelman and Rubin 1992). The portion of variance in y explained by the model was estimated using in-sample Bayesian $R^{2}$ (Gelman et al. 2019). Overall predictive accuracy was assessed by performing five repeats of 10-fold cross-validation (CV), stratified by sex and infection, estimating the distribution of out-of-sample Bayesian $R^{2}$ for each fold, and then aggregating across all folds and repeats.

The CV results were also used to determine the stability of variable inclusions at our selected PIP threshold by recording whether each variable was selected in each fold and repeat. Stability was defined as the proportion of CV splits in which a variable was selected. Given the high correlation among predictors, we defined stably included variables as those selected in at least 70% of CV folds, balancing reproducibility with the inherent variability in variable selection under correlation (Supplementary Fig. 2e). We also calculated Jaccard similarity of selected sets of variables across folds at our chosen PIP threshold, which indicates how often the same variables are selected across folds (Supplementary Fig. 2f).

#### QTL mapping

Quantitative trait locus (QTL) mapping was performed on immune traits in two ways: (i) stratified by infection group and (ii) in a “combined groups” analysis that combines all three infection groups, explicitly tests for significant genotype-by-treatment effects, and weights observations to handle variance heterogeneity.

In the stratified analysis, the fit of a model with covariates and genotype terms was compared with that of a covariates-only model using an *F*-test, yielding a *P*-value reported as $-\log_{10}\;(P)$, hereafter “logP.” In the combined groups analysis, per-marker testing involved comparisons between three weighted linear models:

1. Null model:
  y∼sex+infection
2. Genotype-only model:
  y∼sex+infection+genotype
3. Full model:
  y∼sex+infection+genotype+(genotype×infection)

where y is the trait, sex is a binary variable indicating female if 0 and male if 1, infection is a three-level categorical variable indicating control, SARS-CoV, or SARS-CoV-2, and genotype represents genotype probabilities, as described in the following section (Haley and Knott 1992). Model comparisons were conducted using nested *F*-tests to extract three logPs per marker: i) an overall test comparing models (1) and (3), ii) a marginal genotype test comparing models (1) and (2), and iii) a genotype-by-treatment test comparing models (2) and (3).

Pooling infection groups in the combined analysis introduces potentially disruptive variance heterogeneity (Corty and Valdar 2018). To account for this, all models were fit by weighted least squares with group-specific weights. The weights were derived from infection-group-specific residual variance estimates obtained by first fitting the covariate-only linear model


y∼sex+infection,

and extracting the residuals. The residuals for each infection group *g* were used to calculate a group-specific variance ${\hat{\sigma }}_{g}^{2}$, estimated from their sample variance. Observation-level weights were defined as $w_{i}=1/{\hat{\sigma }}_{g(i)}^{2}$, where g(i) denotes the infection group of individual *i*. These weights were held constant across all markers for a given immune trait.

To assess shared genetic architecture, each locus-trait pair was tested across all groups, and nominal associations (P≤0.05) were counted as evidence of shared effects, since testing at pre-identified positions constitutes targeted hypothesis testing rather than genome-wide discovery.

QTL mapping of viral titer was performed, stratified by infection group, using the two-part model in the R/qtl R package (Broman et al. 2003) to account for its zero-inflated distribution.

##### Genotype probabilities

For the linear model performed at each marker, genotype probabilities were computed using R/qtl (Broman et al. 2003). On autosomes, genotype terms consist of additive effects (add  =P(AB)+2P(BB)) and dominance effects (dom  =P(AB)), and on the X-chromosome, genotype probabilities P(ABf), P(BB), and P(BY) were used as genotype terms as described in Broman et al. (2003). Covariates included in all models were sex, infection (if performing a combined analysis), and pgm (paternal grandmother, describing cross direction, if the marker is on the X-chromosome; Broman et al. 2006).

For example, the following models were fit at each autosomal marker in the combined analysis:

1. Null model:
  y∼sex+infection
2. Genotype-only model:
  y∼sex+infection+add+dom
3. Full model:
  $$\begin{array}{rl} y & ∼sex+infection+add+dom+(add\times infection)\\ & \;+(dom\times infection) \end{array}$$

Analogous models were fit for X-chromosome markers, with X-chromosome genotype probabilities replacing additive and dominance terms, and pgm added as a covariate.

##### Multiple testing correction

Genome-wide adjusted *P*-values were determined by permutation test. A standard permutation approach was applied to stratified analyses: for each trait, the trait was permuted relative to genotype data, a genome scan was performed on the permuted dataset, and the maximum logP value was recorded. This was repeated 1,000 times, and the maximum logP’s were used to fit a Generalized Extreme Value distribution, from which genome-wide *P*-values were derived (Dudbridge and Koeleman 2004; Valdar et al. 2006). This permutation test procedure was modified for combined analyses as follows: (i) permutations were stratified by sex and infection, (ii) weights were recomputed per permutation, and (iii) the maximum logP across all markers and all three tests (overall, marginal genetic, genotype-by-treatment) was recorded per scan as an “omnibus” statistic.

To control for multiple testing across immune traits, experiment-wide *q*-values were obtained by applying the Benjamini-Hochberg False Discovery Rate (FDR) procedure (Benjamini and Hochberg 1995) to the minimum genome-wide adjusted *P*-value for each trait within a given group (stratified control, stratified SARS-CoV, stratified SARS-CoV-2, and combined). This provided *q*-values for the top QTL from each scan; for QTL that were not the most significant in a scan, a linear interpolation function mapped sorted *P*-values to their FDR-adjusted *q*-value. QTL were defined as significant if q<0.05 and suggestive if 0.05≤q<0.1.

##### QTL effect size, sign concordance, and variance decomposition

To define additive and dominance effects by infection group for each autosomal QTL, we performed the linear model,


y∼sex+add+dom,

with add and dom defined from genotype probabilities, as described previously, based on the peak marker. We extracted from this model additive and dominance effect estimates using Type III ANOVA, and *P*-values using a sequential Type I ANOVA. QTL were classified as dominance QTL if no infection group showed a suggestive additive effect (P<0.1) but at least one showed a significant dominance effect (P<0.05) after accounting for additivity.

The effect size for visualization was chosen as the additive effect estimate for additive QTL and the dominance effect estimate for dominance QTL. These effect estimates were square-root transformed, preserving sign, and sign concordance across infections was determined by the direction of effects.

To quantify the relative contribution of additive, dominance, and GxT effects across infection groups, we performed the following linear models for each QTL:


(1) y∼sex+infection

(2) y∼sex+infection+add

(3) y∼sex+infection+add+dom

(4) $$\begin{array}{rl} y & ∼sex+infection+add+dom\\ & \;+(add\times infection)+(dom\times infection) \end{array}$$

and computed partial $R^{2}$ values sequentially, i.e. the contribution of additive effects was estimated relative to a covariate-only model by comparing models (1) and (2), of dominance effects relative to the additive model by comparing models (2) and (3), and of GxT interaction effects relative to the genotype model by comparing models (3) and (4). Partial $R^{2}$ values were estimated from the residual sum of squares (RSS) for each model as:


$$\frac{{\text{RSS}}_{\mathrm{reduced}}-{\text{RSS}}_{\mathrm{full}}}{{\text{RSS}}_{\mathrm{reduced}}}.$$

#### Mediation analysis

To perform context-dependent (or genotype-by-treatment) mediation analysis, we extended the Bayesian model selection framework of bmediatR (Crouse et al. 2022) to the multi-group context. In brief, bmediatR performs Bayesian model selection on a set of $2^{3}=8$ causal models defined by the presence or absence of three directed edges, labeled as *a*, *b*, and *c*, that connect the exposure (*X*), mediator (*M*) and outcome (*Y*) (Supplementary Fig. 3a). Mediation is when edges *a* and *b* are present: complete mediation is when edge *c* is absent, indicating *X* influences *Y* entirely through *M*; partial mediation is when edge *c* is present, indicating both mediated and direct effects of *X* on *Y*. bmediatR also reports colocalization, a specific nonmediation model in which edges *a* and *c* are present, indicating that *X* independently affects both *M* and *Y*. For simplicity, we condense colocalization into the general nonmediation category.

The framework of bmediatR was extended to allow for distinct edges per group (Supplementary Fig. 3b,c) as follows. For *T* groups, group-specific edges are labeled with subscripts to denote group membership, i.e. $a_{t}$, $b_{t}$, and $c_{t}$, and positive evidence for mediation implies that the relevant edges are present within the same group. For example, considering the two-group case, evidence for mediation requires the presence of $a_{1}$ and $b_{1}$, indicating mediation in group 1; $a_{2}$ and $b_{2}$, indicating mediation in group 2; or all four, indicating mediation in both groups (with the presence or absence of $c_{1}$ and $c_{2}$ distinguishing between complete and partial mediation in each group).

The two-group bmediatR model results in six edges with $2^{6}=64$ possible causal models (Supplementary Fig. 3b), and the three-group model results in nine edges with $2^{9}=512$ possible causal models (Supplementary Fig. 3c). Each causal model is categorized independently within treatment group as complete mediation (mediated effect only; $a_{t}$ and $b_{t}$ present, $c_{t}$ absent), partial mediation (mediated and direct effects; $a_{t}$, $b_{t}$, and $c_{t}$ present), or nonmediation (any other combination of edges present). Posterior support is aggregated across models to quantify group-specific and overall evidence for mediation.

This multi-group bmediatR extension was applied to all QTL identified in this cohort of mice: for immune trait QTL reported in this paper, we tested for mediation of weight loss and lung congestion score (CS) by the associated immune trait. For disease-associated loci from Leist et al. (2024) that were nominally associated (P<0.05) with any immune trait, we tested for mediation of the associated disease trait, using the AAC statistic in place of any weight loss trait, by the associated immune trait.

The two-group model (Supplementary Fig. 3b) was used when data were available for only two infection groups (i.e. when the mediator was viral titer and/or the outcome was lung congestion score, which were only available for virus-infected mice), and the three-group model (Supplementary Fig. 3c) was used when data were available for control as well as virus-infected mice. To account for variance heterogeneity in pooled data, we use weighted regression, as allowed for in the original bmediatR package (Crouse et al. 2022), obtaining weights the same way as in the combined groups QTL mapping. Sex and viral titer (if the mediator itself was not viral titer) were controlled for as fixed-effect covariates on both *M* and *Y*.

## Results

### CC006×CC044 ${\text{F}}_{2}$ mice exhibit variable disease severity following coronavirus infection

To investigate immune-mediated mechanisms of coronavirus susceptibility, we analyzed disease severity, viral burden, and lung inflammation in a genetic mapping population infected with SARS-CoV MA15 (hereafter, SARS-CoV; Roberts et al. 2007), SARS-CoV-2 MA10 (hereafter, SARS-CoV-2; Leist et al. 2020), or saline (Fig. 1). As reported previously, a screen of 10 Collaborative Cross (CC) strains identified CC006/TauUnc (CC006) as susceptible and CC044/UncJ (CC044) as resistant to severe disease following infection with SARS-CoV (Leist et al. 2024). Despite their divergent disease outcomes, the two strains exhibited similar viral loads, suggesting that variation in disease severity reflects differences in host immune responses rather than differential susceptibility to infection or impaired control of viral replication.

**Fig. 1.**
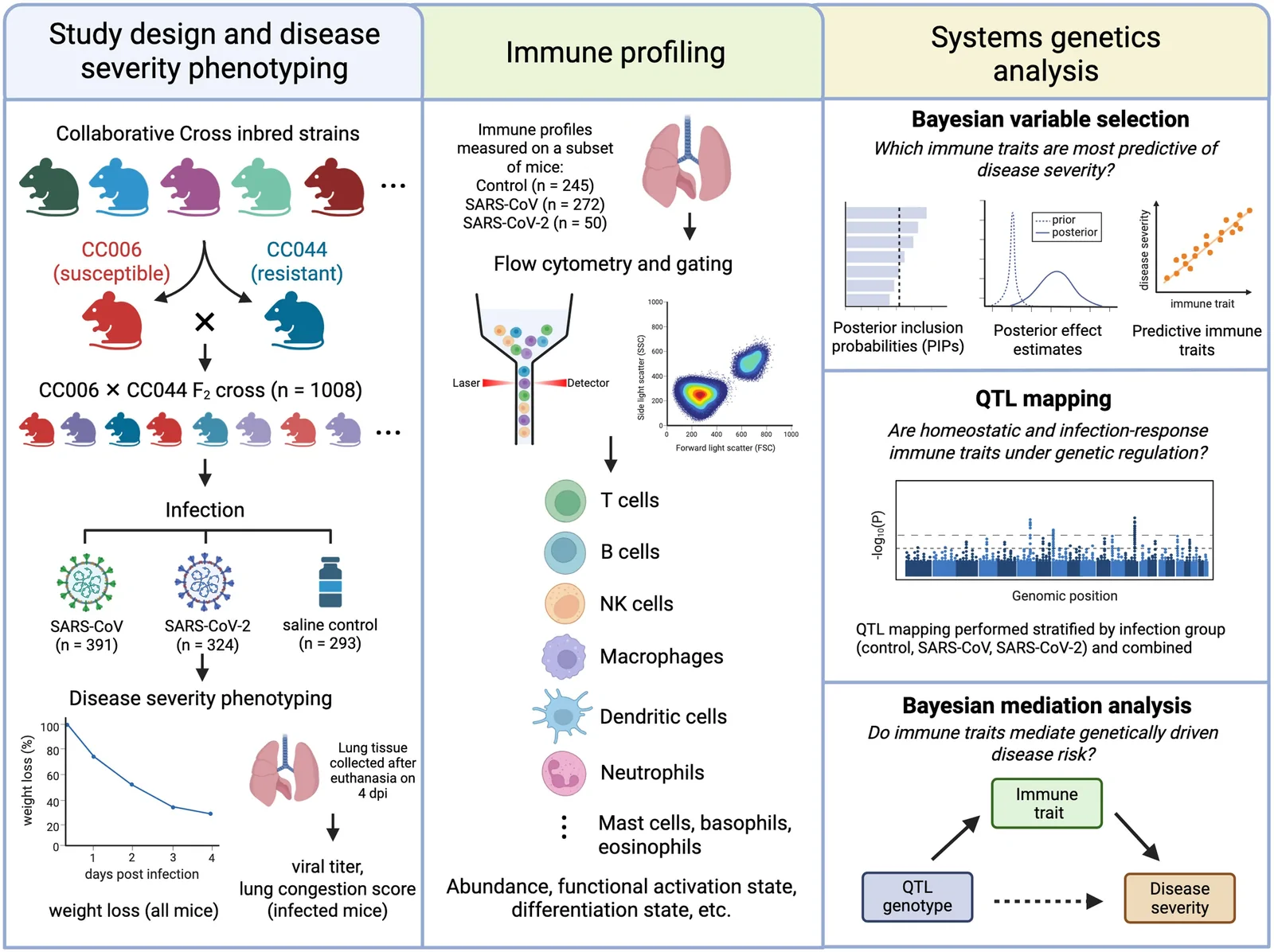
Overview of the study design and statistical framework. Experimental design and statistical analyses used to study immune mechanisms of disease severity. CC006 × CC044 ${\text{F}}_{2}$ mice were mock infected or infected with SARS-CoV MA15 or SARS-CoV-2 MA10, phenotyped for disease severity and lung immune cell composition, and genotyped. Three complementary analyses were then performed: Bayesian variable selection to identify immune predictors of disease severity; infection-stratified and combined QTL mapping to identify genetic loci regulating immune traits at homeostasis and following infection; and context-dependent Bayesian mediation analysis to identify immune traits mediating genetic effects on disease severity. Created in BioRender. Risemberg, E. (2026) https://biorender.com/lc7r800.

To investigate the genetic and immunological basis of these responses, we generated a CC006×CC044 ${\text{F}}_{2}$ mapping population (hereafter, ${\text{F}}_{2}$ mice) comprising 1,008 total mice, each infected with saline (n=293), SARS-CoV (n=391), or SARS-CoV-2 (n=324) (Fig. 1). Mice were monitored daily for weight loss through 4 d post-infection (dpi) and weight loss trajectories were summarized using an area-above-the-curve (AAC) statistic (Fig. 2a-c; Supplementary Fig. 1a; Materials and methods). At 4 dpi, lung tissue was collected to quantify viral titer and pulmonary congestion score (CS) in infected mice (Fig. 2d,e). Weight loss and lung CS in infected mice were reported previously (Leist et al. 2024), and are shown here together with controls and viral titer measurements to establish phenotypic variation in the full mapping cohort.

**Fig. 2.**
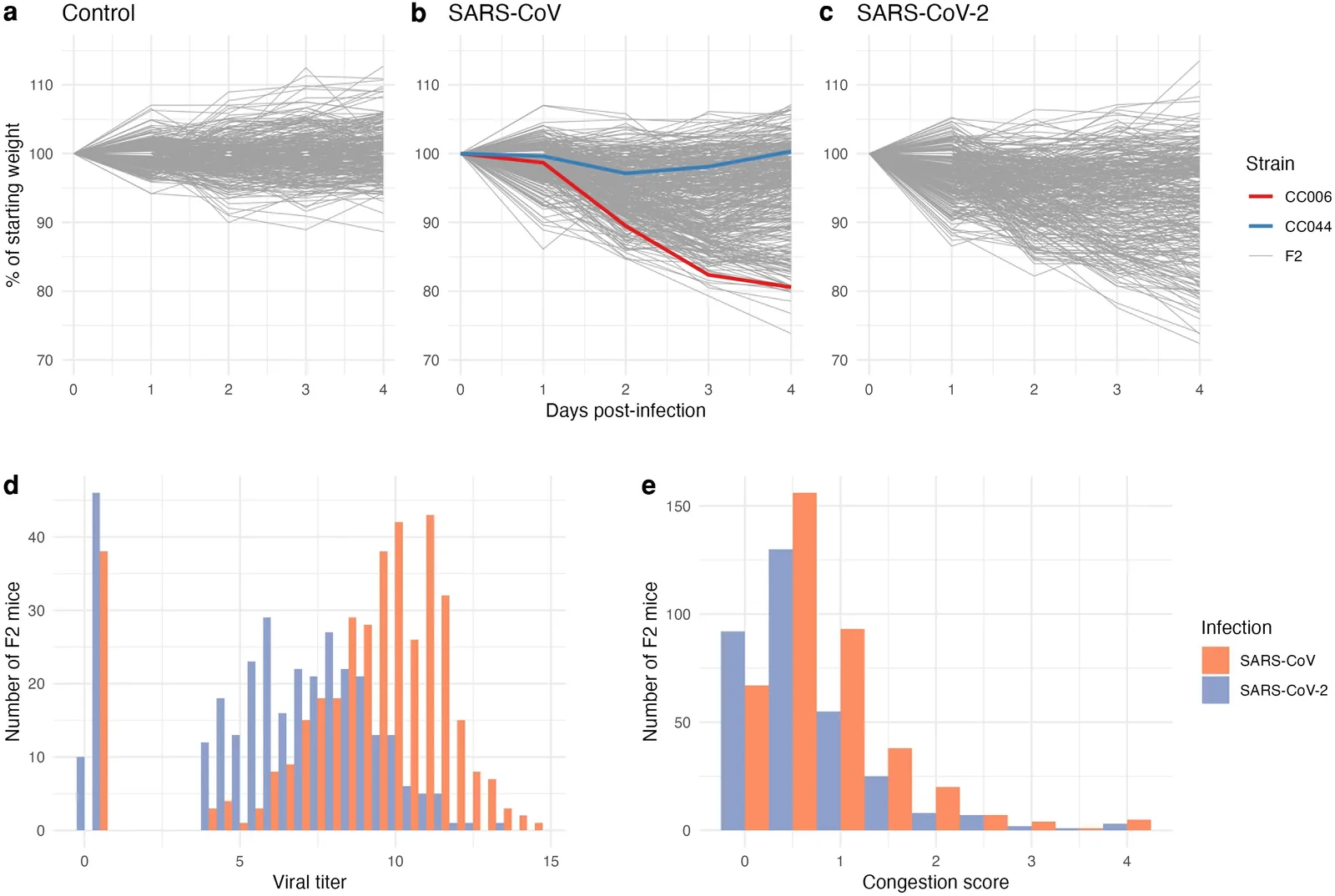
Disease severity traits in ${\text{F}}_{2}$ mice. a) Weight loss in n=293  ${\text{F}}_{2}$ mice after injection with saline. b) Weight loss in n=391  ${\text{F}}_{2}$ mice after infection with SARS-CoV MA15, with average weight loss trajectories of parent strains after infection with the same virus. c) Weight loss in n=324  ${\text{F}}_{2}$ mice after infection with SARS-CoV-2 MA10. d) Log-transformed viral titer and e) lung congestion score (CS) in infected ${\text{F}}_{2}$ mice, collected from lung tissue at 4 dpi. Weight loss and lung CS in virus-infected mice reported previously (Leist et al. 2024). Parental weight loss trajectories are only shown in panel (b) to emphasize that they illustrate weight loss following infection with SARS-CoV MA15 (parental trajectories following infection with saline or SARS-CoV-2 are not available).

Infected ${\text{F}}_{2}$ mice exhibited a broad range of disease severity (Fig. 2), spanning mild to severe weight loss and lung congestion, as well as substantial variation in viral titer following infection, despite parental strains not differing significantly in viral burden. Viral titer was lower in SARS-CoV-2 than in SARS-CoV infection ($P<2.2\times 10^{-16}$, Welch’s *t*-test; Fig. 2d), and was modestly associated with disease severity (Supplementary Fig. 1b,c), consistent with contributions of both viral replication and pulmonary inflammation to disease progression.

### Lung immune profiling reveals correlated and genetically regulated immune variation

To examine immune mechanisms underlying this variation in disease severity, we performed flow cytometry on lung tissue from a subset of mice (n=245 control, n=272 SARS-CoV, and n=50 SARS-CoV-2). The smaller SARS-CoV-2 subset reflects its design for confirmatory rather than exploratory analyses. Using three antibody panels, we quantified 105 immune traits capturing variation in lymphoid and myeloid composition, activation state, MHC II expression, and trafficking receptor expression (Supplementary Fig. 1d; Supplementary Table 2; Materials and methods). Immune traits had large batch and infection effects, with modest sex and polygenic effects (Supplementary Fig. 1e)—SNP-based heritability ($h_{\mathrm{SNP}}^{2}$; Yang et al. 2017) estimates ranged from 0 to 50% (mean 3%), with 15 traits having $h_{\mathrm{SNP}}^{2}>5\%$ (Supplementary Table 2; Materials and methods).

Importantly for subsequent analyses, immune traits exhibited extensive multicollinearity (Fig. 3a; Supplementary Fig. 4), with multiple correlation coefficients ($R^{2}$) ranging from 0.65 to 0.99, and VIFs indicating multicollinearity for 90 out of 105 traits (Supplementary Table 2; Materials and methods). Principal components analysis revealed clear clustering by infection group (Fig. 3b), and immune profiles distinguished infection status with high accuracy (mean cross-validated accuracy 98.8%±0.005%; Materials and methods). Together, these data provide a foundation for mapping genetic regulation of immune responses, identifying immune predictors of disease severity, and defining pathways consistent with immune mediation of genetic risk.

**Fig. 3.**
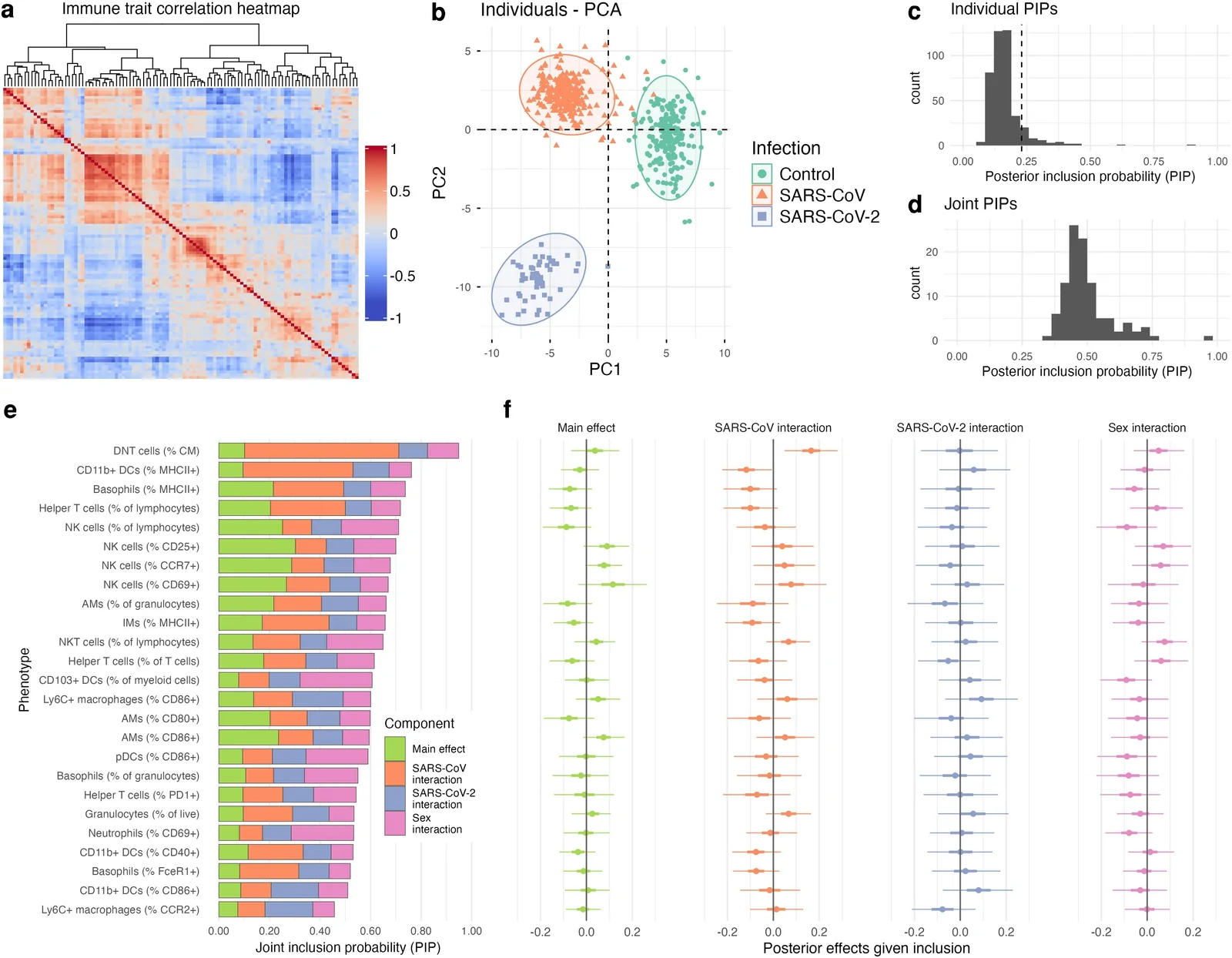
Immune traits in ${\text{F}}_{2}$ mice. a) Heatmap showing correlation of immune traits in ${\text{F}}_{2}$ mice (labeled version in Supplementary Fig. 4). Hierarchical clustering is based on Euclidian distance of the correlation matrix. b) Principal component analysis (PCA) plot of individuals based on 105 imputed immune traits. Ellipses represent multivariate normal confidence regions around each group mean based on a multivariate *t*-distribution. c) Histogram of individual (per-predictor) posterior inclusion probabilities (PIPs), with the inclusion threshold of 0.23 shown by a dashed vertical line, and d) joint (per-immune trait) PIPs. e) Joint PIPs for the 25 predictive traits, defined by having any main or interaction effect (i) exceeding the individual PIP threshold and (ii) being stably included across CV folds. Colors represent relative sizes of individual PIPs for main and interaction effects as a portion of the joint PIP for each trait. f) Posterior distributions of effect sizes given inclusion. Dots represent mean effect size conditional on inclusion in the model (i.e. $\beta_{j}$ when $\gamma_{j}=1$), thin lines represent 95% credible intervals, and thick lines represent 50% credible intervals. Because sex is coded as a binary variable (female = 0, male = 1), sex-interaction effects represent the additional, male-specific deviation in immune trait effects on weight loss, relative to females.

### Specific immune cell traits are associated with weight loss

To determine which immune traits associated most strongly with disease severity, we applied Bayesian variable selection to model weight loss (AAC) as a function of sex, infection group, and 105 correlated immune traits, totaling 420 predictors under selection when allowing for trait-by-sex and trait-by-infection interactions (Materials and methods; Appendix B). We used stochastic search variable selection (SSVS; George and McCulloch 1993, 1997), which applies a “spike-and-slab” prior (Supplementary Fig. 2a) to shrink estimated effects toward 0, encouraging sparsity and providing posterior inclusion probabilities (PIPs) for each predictor. We imputed missing immune data simultaneously with variable selection so that results accounted for uncertainty in missing data imputation.

Median in-sample Bayesian $R^{2}$ was 0.48 (Supplementary Fig. 2c), indicating about half of the variance in weight loss was explained by the model. Five repeats of 10-fold cross-validation (CV) produced a median out-of-sample Bayesian $R^{2}$ of 0.38 (Supplementary Fig. 2d), demonstrating that although predictive accuracy is not our primary goal, the model generalized well to unseen data. As expected, infection group exhibited strong posterior evidence of association with weight loss, with both SARS-CoV and SARS-CoV-2 infection producing greater weight loss than controls ($\beta_{\mathrm{SARS}-\mathrm{CoV}}=0.77$ (95% CI: 0.31 to 1.21); $\beta_{\mathrm{SARS}-\mathrm{CoV}-2}=0.95$ (95% CI: −0.02 to 1.88)). Sex also showed evidence of a modest independent effect on disease severity after accounting for immune traits, with males exhibiting less weight loss than females ($\beta_{\mathrm{sex}}=-0.34$ (95% CI: −0.49 to −0.19)).

Because the standard 0.5 PIP threshold is overly conservative under multicollinearity (Ghosh and Ghattas 2015), we established an empirical inclusion threshold for immune predictors of 0.23 using permutation (Fig. 3c; Materials and methods). CV results were also used to identify stably included variables (Materials and methods). Thirty-two predictors, representing main and interaction effects from 25 immune traits (hereafter, predictive traits), exceeded the empirical inclusion threshold and were stably included in CV (Fig. 3e; Supplementary Table 2). Joint PIPs, representing the posterior probability that any effect (main or interaction term) for a trait is included, ranged from 0.36 to 0.95, and from 0.46 to 0.95 for predictive traits (Fig. 3d). Predictive traits spanned multiple immune compartments, including various subsets of T cells, macrophages, dendritic cells (DCs), natural killer (NK) cells and basophils, and described abundance, activation and differentiation state of these subsets (Fig. 3e).

Effect sign concordance between SARS-CoV and SARS-CoV-2 was established as traits with consistent effect direction across viruses in ≥80% of posterior samples (Materials and methods). Predictive traits showed largely concordant effects across both viruses, with 19 of 25 traits exhibiting consistent effect direction (Supplementary Table 2). CD11b^+^ DCs (% MHC II^+^) had the lowest sign concordance at 50%, indicating that the posterior mass included substantial probability of a same-direction effect, particularly due to uncertainty in the estimation of the SARS-CoV-2-interaction effect (Fig. 3f). Across predictive traits, conditional 95% credible intervals for SARS-CoV-2 interaction effects were larger than those for main, SARS-CoV interaction and sex interaction effects ($P=3.0\times 10^{-8}$, Wilcoxon signed rank test; Fig. 3f), likely owing to the smaller sample size of immune phenotyped SARS-CoV-2-infected mice.

Mean virus-specific immune trait effects were estimated as the sum of the posterior means of the main effect and the virus-interaction effect, each conditional on inclusion in the model (Supplementary Table 2; Materials and methods). A positive effect direction indicates increased weight loss (consistent with a deleterious effect on disease severity) and a negative effect direction indicates decreased weight loss (consistent with a protective effect on disease severity). The trait with the highest PIP, central memory (CM) double negative (DN) T cells (Fig. 3e), was estimated to have a large deleterious effect on weight loss after SARS-CoV infection (Fig. 3f; Supplementary Table 2). Relative abundance of helper T cells and NK T cells were estimated to have protective and deleterious effects, respectively, in both infection groups (Fig. 3f; Supplementary Table 2). MHC II expression on basophils and interstitial macrophages (IMs) was estimated to have a protective effect after both infections, while that of CD11b+ DCs had a protective effect in SARS-CoV infection and a small deleterious effect in SARS-CoV-2 infection (Fig. 3f; Supplementary Table 2). Relative abundance and CD80 expression of alveolar macrophages (AMs) were estimated to have a protective effect in both infections, while CD86-expressing AMs had a deleterious effect in both infections (Fig. 3f; Supplementary Table 2). While increased relative abundance of NK cells was estimated to have a protective effect on weight loss, increased NK cell activation and mobilization (as defined by the proportion of NK cells expressing CD25, CD69, or CCR7) was estimated to have a deleterious effect (Fig. 3f; Supplementary Table 2).

Across predictive traits, posterior mean sex-interaction effects were similar in magnitude to posterior mean virus-interaction effects (P=1.00, paired Wilcoxon signed rank test; Fig. 3f), showing that immune effects vary by sex to a similar degree as they vary by infection status. Sex-interaction effects for predictive immune traits were predominantly negative (18 of 25 traits; Supplementary Table 2), indicating that immune trait associations with weight loss were generally attenuated in males relative to females.

### Immune traits are under genetic regulation

To identify genetic determinants of immune variation in the ${\text{F}}_{2}$ mice, we mapped quantitative trait loci (QTL) for 105 lung immune cell traits in infection-stratified and combined analyses. To increase power and explicitly test for context-dependent effects in the combined analysis, we implemented a multi-group mapping framework that jointly models immune traits across infection conditions, tests for genotype-by-treatment (GxT) interaction effects, and uses weighted least squares to account for variance heterogeneity introduced by pooling infection groups—a substantial concern in this dataset, as 82 of 105 immune traits exhibited significant variance heterogeneity across infection groups (P<0.05, Levene’s test; Supplementary Table 2; Materials and methods).

We identified 68 significant or suggestive locus-trait associations, corresponding to 41 distinct QTL named *Irq* (Immune response QTL) 1 through 41 (Fig. 4a; Supplementary Table 3). Eighteen of these loci were only identified in the combined analysis, consisting of small-effect size loci that were underpowered in stratified scans. These QTL explained less phenotypic variance on average (3.7%) than QTL identified by the stratified approach (19.2%; Welch’s two-sample *t*-test, $P=8.1\times 10^{-8}$). Specifically, pooling data and regressing on both a genotype and GxT term at each marker allows us to detect moderate same-direction effects with the main genetic effect term, and moderate opposite-direction effects with the GxT interaction term. *Irq12*, for example, captures only nominally significant infection-specific effects (P=0.03 in control mice, P=0.002 in SARS-CoV-infected mice) but has a highly significant interaction term ($P=8.0\times 10^{-4}$) that reached genome-wide significance in the combined analysis. Since both the stratified and combined analyses identified QTL not detected by the other approach, a combination of approaches is most appropriate in such a treatment-control context.

**Fig. 4.**
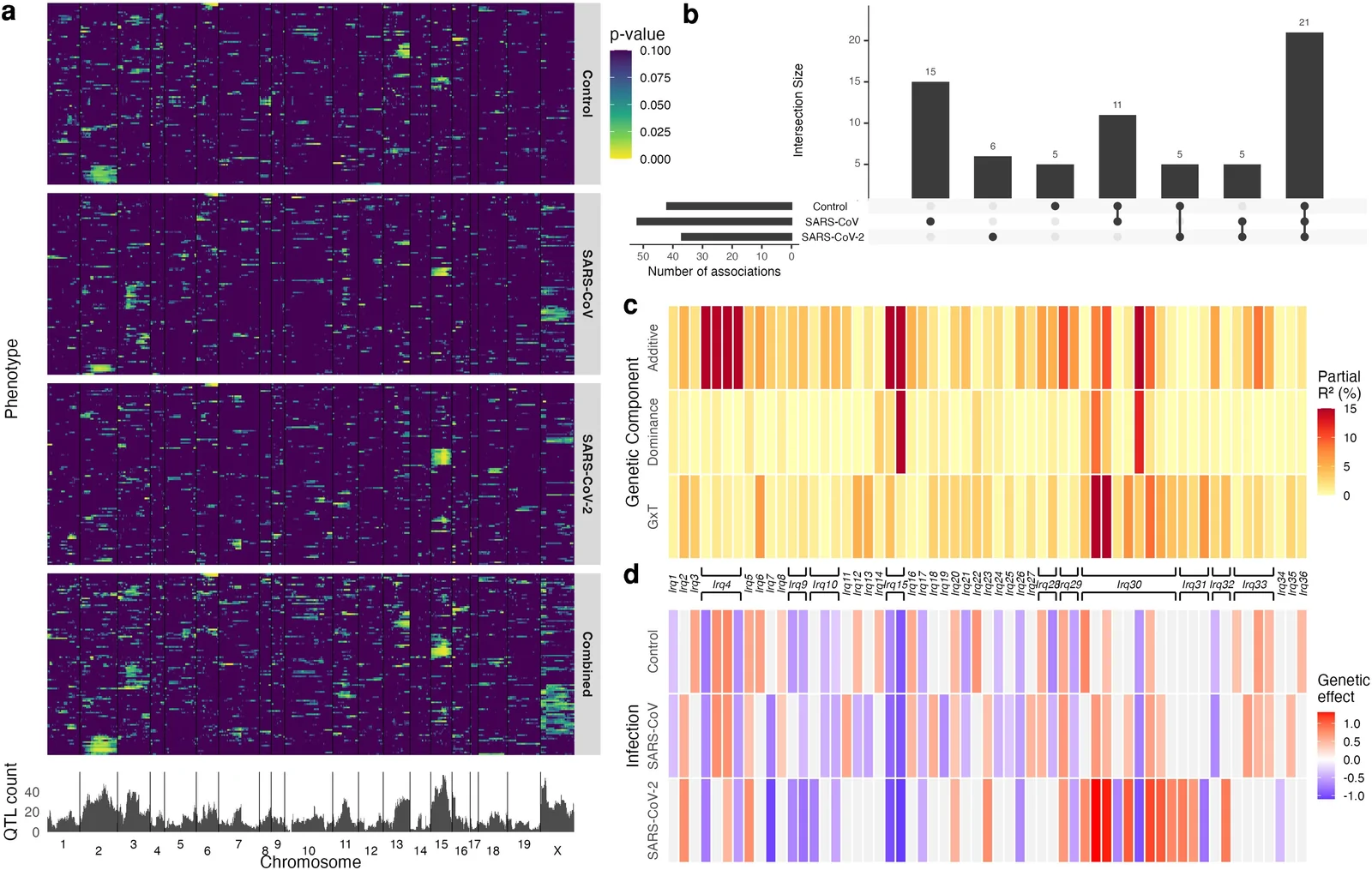
Genetic architecture of immune trait QTL across infection groups. a) Genome-wide distribution of significant locus-trait associations across 105 immune traits and across stratified and combined analyses. The traits on the *y*-axis are ordered according to hierarchical clustering based on similarity of QTL scans to highlight shared genetic architecture (Supplementary Table 2). Color indicates strength of association in terms of genome-wide associated *P*-value (capped at 0.1 for visualization). Histogram rug at bottom of plot shows density of significant marker-trait associations (P<0.05) across traits and groups. b) Upset plot showing overlap of QTL associations across infection groups. QTL are counted as present in a group if they showed nominal association (P<0.05) at positions reaching experiment-wide significance (q<0.1) in at least one group. c) Variance partitioning of genetic effects at each locus-trait association for autosomal QTL. Heatmap shows partial $R^{2}$, the proportion of residual variance explained by additive, dominance, and genotype-by-treatment effects, sequentially, after accounting for sex and infection. d) Additive genetic effect sizes across infection groups for autosomal QTL. Heatmap shows standardized genetic effect sizes (square-root transformed coefficients) for each association tested in each infection group. Red and blue indicate CC044 alleles are associated with increased and decreased trait values, respectively. Dominance effects are shown instead of additive effects for dominance QTL: *Irq19*, *Irq22*, and the first column of *Irq30* (association with CCR2^+^ Ly6C^+^ macrophages), for which red and blue indicate that CC006/CC044 heterozygotes are associated with increased and decreased trait values, respectively. Gray indicates nonsignificant associations (P≥0.05). Brackets indicate QTL names; for QTL with multiple associated traits, traits are shown in the same order as they are listed in Supplementary Table 3.

Despite the smaller subset of immune phenotyped SARS-CoV-2-infected mice, multiple associations were detected in this cohort (Fig. 4a,b). To assess shared genetic architecture, we tested each locus-trait pair across all groups and counted nominal associations (P≤0.05) as evidence of shared effects (Materials and methods). This revealed both shared and infection-specific architecture: about 38% of locus-trait associations were specific to a single infection group (5 in control, 15 in SARS-CoV, and 6 in SARS-CoV-2), 31% were present in all three conditions, and the remainder were shared across two of the three groups (Fig. 4b).

Genetic effects at immune QTL were predominantly additive, with additional variance explained by dominance and GxT effects (Fig. 4c; Materials and methods). Additive effects explained, on average, 5% of residual variance after accounting for covariates (range 0-44%) (Fig. 4c). Three associations were driven by dominance effects—*Irq19*, *Irq22*, and the association of *Irq30* with CCR2^+^ Ly6C^+^ macrophages—highlighting the importance of modeling both additive and dominance effects in genetic mapping (Fig. 4c). GxT effects explained a significant amount of variation remaining after accounting for additive and dominance effects (0–26%, mean 3%), supporting the conclusion that many genetic effects are infection-specific or vary across infection groups (Fig. 4c). Most associations with effects across infection groups were sign concordant (Fig. 4d). QTL identified in the SARS-CoV-2 cohort had larger effect sizes than QTL identified in the control or SARS-CoV cohorts ($P=1.0\times 10^{-4}$), reflecting an expected bias towards large effect QTL in smaller samples (Fig. 4d; Göring et al. 2001).

Viral titer required a distinct modeling strategy from other immune traits due to its distributional properties. Since log-transformed titer exhibits a zero-inflated distribution (Fig. 2d), we used a two-part model (Broman et al. 2003), which enables the detection of loci influencing both the binary (presence/absence) and continuous components of a trait. This mapping was performed stratified by infection group. One QTL (*Irq7*) reached genome-wide significance for viral titer, the effect of which is driven by continuous variation, with mice homozygous for the CC044 allele having lower titer (Fig. 4d; Supplementary Table 3). Despite previously finding no differences in viral loads between the two parent strains (Leist et al. 2024), this QTL suggests there might be a role for a host-driven change in viral clearance or viral replication in the resistance seen in CC044.

### Immune traits are mediators of genetically driven disease risk

Having established that immune traits are both associated with disease severity and subject to genetic regulation, we next performed mediation analysis to test for evidence that immune traits mediate genetic risk. Mediation analysis is a type of causal inference designed to evaluate whether the effect of an exposure (*X*) on an outcome (*Y*) is explained by a third, mediator variable (*M*), thereby suggesting causal pathways that may underlie genetic risk. To perform context-dependent mediation analysis, we extended bmediatR (Crouse et al. 2022), a Bayesian model selection approach to mediation analysis, to the multi-group setting (Materials and methods).

In the original bmediatR framework, mediation is evaluated through three causal paths: the effect of a genotype or other exposure (*X*) on a potential mediator (*M*; path *a*), the effect of *M* on the outcome (*Y*; path *b*), and the direct effect of *X* on *Y* (path *c*) (Supplementary Fig. 3a). Mediation occurs when paths *a* and *b* are present—complete mediation when path *c* is absent, indicating that *X* influences *Y* entirely through *M*, and partial mediation when path *c* is present, indicating both mediated and direct effects of *X* on *Y*.

Our extension allows these three causal paths to differ between experimental contexts. When measurements are only available in virus-infected mice, paths may differ between the two viruses, resulting in six group-specific paths: $a_{1}$, $b_{1}$, and $c_{1}$ in SARS-CoV-infected mice, and $a_{2}$, $b_{2}$, and $c_{2}$ in SARS-CoV-2-infected mice (Supplementary Fig. 3b). When measurements are available in mock- and virus-infected mice, paths may differ between all three groups, resulting in nine group-specific paths: $a_{1}$, $b_{1}$, and $c_{1}$ in control mice, $a_{2}$, $b_{2}$, and $c_{2}$ in SARS-CoV-infected mice, and $a_{3}$, $b_{3}$, and $c_{3}$ in SARS-CoV-2-infected mice (Supplementary Fig. 3c). We computed posterior probabilities for all $2^{6}=64$ possible causal models in the two-group case, or all $2^{9}=512$ possible causal models in the three-group case. Defining mediation as the presence of both the X→M and M→Y effects in the same group, we summarized evidence for mediation patterns within and across infection contexts. Compared with a stratified approach (performing standard mediation analysis in each group), GxT mediation analysis enables inference on context-specific mediation patterns within a single probabilistic framework while borrowing information across groups.

We applied this mediation approach to triplets of data consisting of genotypes for immune and disease trait QTL (Leist et al. 2024) as exposures (*X*), immune traits (including viral titer) as candidate mediators (*M*), and weight loss and lung CS as outcomes (*Y*) (Materials and methods). Mediation analysis on these triplets evaluates whether the genetic locus affects disease severity through effects on immune variation. We identified 43 locus-immune trait pairs (corresponding to 25 loci and 32 immune traits) with substantial evidence, defined as log-transformed Bayes factor (BF) greater than 0.5 (Jeffreys 1961), for complete or partial mediation of weight loss or lung congestion (Supplementary Table 4). We found that genetic loci with evidence for mediation were often shared across both viruses, although the specific immune trait mediator frequently varied: about half (12) of the 25 loci showed mediation in both infections, but only four of these loci had the same mediator across infections.

Evidence for mediated effects at the conserved disease locus *HrS43*, originally reported in Leist et al. (2024), illustrates how shared genetic susceptibility can act through distinct immune mechanisms across infections. The effect of *HrS43* on weight loss showed evidence of mediation by central memory (CM; CD62L^+^ CD44^+^) double negative (DN) T cells in SARS-CoV infection (Fig. 5a-d) but by activated (CD86^+^) inflammatory monocytes in SARS-CoV-2 infection (Fig. 5e-h). Specifically, in SARS-CoV-infected mice, the *HrS43* locus is nominally associated with the proportion of DN T cells exhibiting a CM phenotype (Fig. 5b), which is in turn associated with increased weight loss (Fig. 5c), with strong support for complete mediation (Fig. 5d; Supplementary Table 4). In contrast, in SARS-CoV-2-infected mice, *HrS43* is associated with the proportion of inflammatory monocytes expressing activation marker CD86 (Fig. 5f), which in turn is associated with increased weight loss (Fig. 5g), with some evidence for complete mediation (Fig. 5h; Supplementary Table 4).

**Fig. 5.**
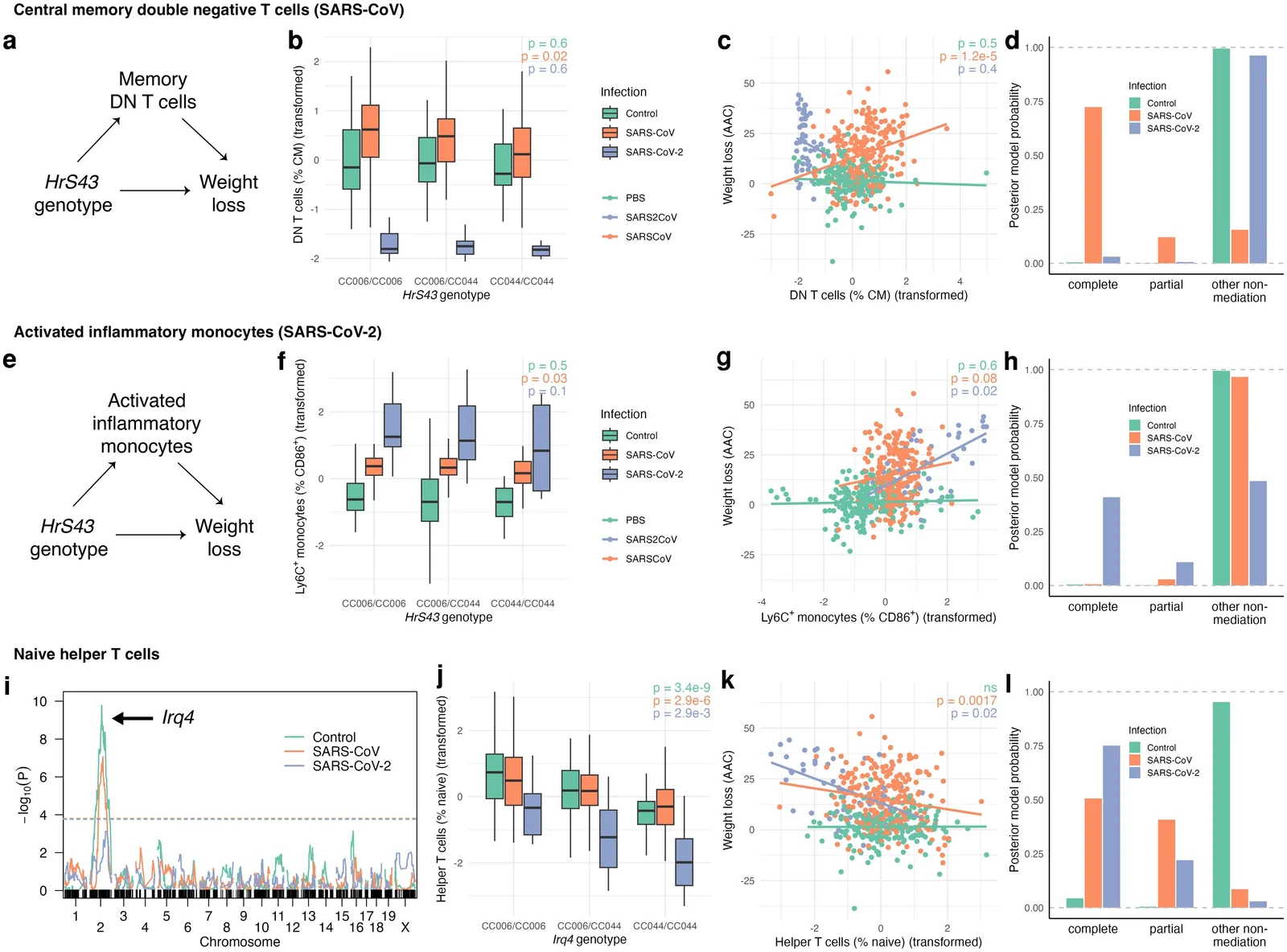
Immune-mediated genetic effects on coronavirus disease severity. Proportional immune traits were arcsine transformed then centered and scaled prior to analysis and visualization. a) Schematic of mediation by central memory (CM) double negative (DN) T cells. b) Effect of *HrS43* genotype on CM DN T cells. c) Association between CM DN T cells and weight loss. d) Posterior probability of mediation of *HrS43*-induced weight loss by CM DN T cells. e) Schematic of mediation by activated (CD86^+^) inflammatory monocytes. f) Effect of *HrS43* genotype on activated inflammatory monocytes. g) Association between activated inflammatory monocytes and weight loss. h) Posterior probability of mediation of *HrS43*-induced weight loss by activated inflammatory monocytes. i) Stratified genome scan of naïve helper T cells. j) Effect of *Irq4* on proportion of naïve helper T cells. k) Association between naïve helper T cells and weight loss. l) Posterior probability of mediation of *Irq4*-induced weight loss by naïve helper T cells.

In contrast to *HrS43*, *Irq4* acts through immune pathways that are conserved across both infections. *Irq4* is associated with the proportion of naïve T cells (Fig. 5i,j), which in turn is associated with decreased weight loss (Fig. 5j), with strong evidence for mediation in both infection groups (Fig. 5l; Supplementary Table 4). Increased proportions of naïve T cells were associated with reduced disease severity, consistent with a protective role for preserved naïve T cell compartments during infection. These findings illustrate that, alongside virus-specific immune mechanisms, some genetic loci may influence coronavirus disease severity through conserved immunological pathways.

## Discussion

Here we combine Bayesian variable selection, QTL mapping, and context-dependent mediation analysis to dissect genetic regulation of immune traits and their contribution to disease severity. Bayesian variable selection identified a robust set of immune predictors of weight loss with largely consistent effect directions across SARS-CoV and SARS-CoV-2 infection, indicating broadly shared immune protection and pathogenesis. In contrast, QTL mapping of immune traits revealed a mixture of shared and infection-specific genetic architecture, with approximately one third of immune QTL detected in only a single infection group.

Context-dependent mediation analysis further revealed that several QTL have mediated effects on disease severity in both viruses, but the specific immune mediators often vary between viruses. This pattern suggests that host susceptibility loci can influence disease severity across infections while acting through distinct, virus-dependent immune mediators. Consistent with this model, we find evidence that *HrS43*, a disease-associated locus conserved between mouse and human and across the sarbecovirus subgenus (Leist et al. 2024), acts through different immune traits in each infection: central memory double negative T cells in SARS-CoV, and activated inflammatory monocytes in SARS-CoV-2 (Fig. 5a-h; Supplementary Table 4). A notable exception to this pattern was *Irq4*, whose effects on disease severity showed evidence of mediation by naïve T cell subsets in both infections, indicating a conserved immune pathway across viruses (Fig. 5i-l; Supplementary Table 4).

### Methodological considerations and inferential scope

Our statistical framework for the integrative analysis of genotypes, immune traits and disease traits has several strengths and limitations. In our Bayesian variable selection analysis, importance is defined in terms of posterior inclusion probability (PIP) and stability across cross-validation folds. Because immune traits measured by flow cytometry are highly correlated, posterior probability mass is often distributed across multiple competing models, reducing the marginal inclusion probability of any single trait despite shared biological signal. Consequently, conservative PIP thresholds may exclude traits that nonetheless contribute to disease severity. Conversely, traits selected by the model should not be interpreted as causal or necessarily of large effect: PIPs quantify evidence that an effect is nonzero, not its magnitude or mechanistic importance. Both selected and nonselected traits should therefore be interpreted in the context of prior biological knowledge. In addition, PIPs depend on prior assumptions about effect sizes and model sparsity and can be sensitive to hyperparameter choice. To address this concern, we assessed robustness across a range of reasonable hyperparameters and found that, while the mean of the PIP distribution varied, the overall shape and relative ranking of traits within the distribution remained relatively consistent (Materials and methods; Appendix B).

Mediation analysis also has limitations. Since it requires genotype—immune trait—disease trait triplets, we can only test for locus-specific mediation when genetic regulation of the immune trait is detectable, and only for disease variation attributable to the detected loci. Failure to detect mediation thus does not imply that an immune trait is uninvolved in disease, but rather that mediation by the specified locus could not be established. These constraints are particularly relevant for the SARS-CoV-2 cohort, for which immune profiling was available for a limited subset of animals (n=50), limiting QTL detection.

In addition, interpretation relies on assumptions about causal order and direction as well as the absence of unmeasured confounding. Reactive or reverse mediation, where disease severity influences immune composition, is biologically plausible in viral disease but difficult to distinguish statistically (MacKinnon et al. 2007). Temporal precedence—that the change in mediator precedes the change in outcome—is also an important condition of causality (Kendall et al. 2017). The outcome in this study summarizes weight loss trajectories from days 1–4, whereas immune traits were measured on day 4; thus, immune measurements serve as imperfect proxies for immune variation over the course of infection, which may limit strict directional causal interpretation but does not preclude biological relevance.

Nonetheless, because our mediation analysis is based on Bayesian model selection, it offers important advantages. In our framework, evidence for mediation reflects support for a joint model relative to alternative models, rather than requiring each component association to exceed a fixed significance threshold, as in the commonly used “causal steps” approach to mediation (Baron and Kenny 1986). This framework explicitly captures uncertainty in the inferred mediating relationship and allows evaluation of competing causal explanations. For example, CCR2^+^ inflammatory (Ly6C^+^) monocytes were supported as mediators of SARS-CoV-2 disease despite weak marginal associations with weight loss, accompanied by substantial posterior uncertainty across mediation and nonmediation models (Supplementary Table 4).

Pooling data across infection groups introduces variance heterogeneity that can reduce power and inflate false positives if unmodeled (Corty and Valdar 2018). We address this in both combined QTL mapping and context-dependent mediation analysis using weighted regression, assigning lower weight to noisier observations. Although unweighted analyses increased detection of QTL and candidate mediators, weighting reduced support for a subset of these signals, suggesting that some associations in unweighted models may reflect spurious effects driven by variance heterogeneity.

Interpretation of SARS-CoV-2-specific results is further shaped by features of the experimental design. Although the overall SARS-CoV-2 infection cohort was similar in size to other groups, immune profiling was performed on a smaller subset of animals, reflecting its design for confirmatory analyses. Nevertheless, several immune QTL were identified in this cohort, with a bias towards large effect QTL as expected under limited power (Fig. 4d; Göring et al. 2001). In addition, although control and SARS-CoV immune profiles were generated across multiple batches, all SARS-CoV-2 immune profiles were generated in a single experimental batch, resulting in confounding between batch and infection status. This confounding necessitated conservative modeling choices and, as a result, a fuller treatment of infection effects on immune traits was beyond the scope of this study; however, because ${\text{F}}_{2}$ mice are outbred and littermates were randomized across batches, genetic effects and genotype-by-infection effects remain estimable (Materials and methods).

Finally, interpretation of immune QTL should consider that some associations may reflect genetic variation in antibody recognition rather than biologically meaningful differences in immune cell abundance. For example, the highly significant *Irq15* QTL for the proportion of NK and NK T cells expressing NK1.1 (Supplementary Table 3) is consistent with the well-characterized strain-specific nature of NK1 antibody reactivity, determined by allelic variation within the NK gene complex on chromosome 6 (Carlyle et al. 2006), and therefore likely reflects differential antibody binding rather than differential NK1.1 expression. Nonetheless, this QTL serves as a positive control and validates our general QTL mapping approach.

In contrast, *Irq4*, which regulates the proportion of naïve and central memory helper and cytotoxic T cells (Supplementary Table 3), maps directly over *Cd44*, the marker used to distinguish naïve (CD44^-^ CD62L^+^) and central memory (CD44^+^ CD62L^+^) T cell populations (Supplementary Table 1). The association between naïve helper T cells and disease severity (Fig. 5k) makes a purely technical explanation less likely, suggesting that, if *Cd44* is causal for this QTL, it reflects genuine biological variation in *Cd44* expression and function rather than solely differential antibody recognition. Indeed, *Cd44* is a functional regulator of T-cell activation, migration, and memory differentiation (Baaten et al. 2010), making it a plausible candidate gene.

### Interpretation of immunological findings

Taken together, the immune traits identified by variable selection and mediation analyses define a focused set of candidates with consistent statistical support for involvement in coronavirus disease pathogenesis. We highlight a small number of representative examples that illustrate two central themes emerging from this study: (i) virus-dependent mediation of shared genetic risk loci, and (ii) convergence on MHC II expression and myeloid subsets that are broadly consistent with observations in human SARS and COVID-19.

A key example of virus-dependent mediation is provided by the conserved disease-associated locus *HrS43* (Fig. 5a-h). In SARS-CoV-infected mice, central memory double negative (DN) T cells are predictive of weight loss with a large deleterious effect, and mediate *HrS43*-driven weight loss (Fig. 5a-d; Supplementary Table 2, Supplementary Table 4). Although canonical memory T cells persist after an infection is resolved, pre-activated memory-like DN T cells exist and have been associated with respiratory virus infection (Neyt et al. 2016; Velikkakam et al. 2022). In contrast, the same locus affected SARS-CoV-2 disease severity through activated (CD86^+^) inflammatory monocytes (Fig. 5e-h; Supplementary Table 2, Supplementary Table 4). This is consistent with research associating an accumulation of inflammatory monocytes in the lungs with severe SARS and COVID-19 (Nicholls et al. 2003; Channappanavar et al. 2016; Chua et al. 2020; Liao et al. 2020; Dorward et al. 2021; Grant et al. 2021; Chen et al. 2022). Although we do not find evidence for mediation by activated inflammatory monocytes in SARS-CoV disease, they are predictive of weight loss with a deleterious effect in both infection groups (Supplementary Table 2).

*Irq10* provides another example of a shared locus which showed evidence of mediation by virus-specific immune traits. This locus has an effect on lung congestion that is mediated by CD69^+^ cytotoxic T cells in SARS-CoV, and an effect on weight loss that is mediated by CD25^+^ cytotoxic T cells in SARS-CoV-2 (Supplementary Table 4). The fact that CD69 and CD25 are both activation markers suggests the possibility that we are capturing related traits functioning in a single pathway across viruses.

*Irq30* has effects on disease severity with evidence of mediation by distinct traits in each virus, illustrating the complexity of potential causal pathways at some loci. *Irq30* is associated with nine monocyte/macrophage-related traits across infection groups, including overall abundance and chemokine receptor (CCR2) expression of both inflammatory (Ly6C^+^) and patrolling (Ly6C^–^) monocytes (Supplementary Table 3). We find evidence that *Irq30*-driven inflammatory monocyte abundance mediated SARS-CoV disease with a deleterious effect on lung congestion (Supplementary Table 4), consistent with the observed association between inflammatory monocyte abundance and severe disease in humans (Nicholls et al. 2003; Liao et al. 2020; Grant et al. 2021; Chen et al. 2022).

Mediation by CCR2 expression reflects a more complex pattern. Although CCR2 is canonically associated with inflammatory monocytes (Li YH et al. 2022), *Irq30* affects CCR2 expression on both inflammatory and patrolling monocytes in our study (Supplementary Table 3). We find evidence that CCR2 expression on patrolling monocytes mediates SARS-CoV disease with a deleterious effect, and that CCR2 expression on inflammatory monocytes mediates SARS-CoV-2 disease with a protective effect (Supplementary Table 4). The opposing effects of CCR2 expression on patrolling and inflammatory monocytes illustrate the distinct nature of these cell types, with inflammatory monocytes promoting inflammation and patrolling monocytes being primarily regulatory in function (Murray 2018).

In addition, abundance of inflammatory monocytes and CCR2 expression on inflammatory monocytes, while closely related traits, exhibit opposing associations with disease severity (Supplementary Table 4). This distinction may reflect the fact that CCR2 expression captures recruitment potential, whereas monocyte abundance reflects the realized accumulation of cells in lung tissue. Considering prior studies linking both inflammatory monocyte abundance and CCR2-dependent recruitment to severe coronavirus disease (Zhou Z et al. 2020; The GenOMICC Investigators 2021), these results appear contradictory at face value. However, experimental work has also demonstrated a protective role for CCR2-dependent monocyte infiltration during acute infection, with CCR2-deficient mice exhibiting higher viral loads and exacerbated inflammation (Vanderheiden et al. 2021). In addition, reduced CCR2 expression on monocytes has been observed in individuals recovered from severe COVID-19 compared to moderate disease, supporting a role for protection at baseline (Liechti et al. 2022). Although all measurements in our study were collected at a single time point, these findings are most consistent with a model in which CCR2-mediated recruitment supports early antiviral responses, whereas sustained accumulation of inflammatory monocytes contributes to immunopathology. Under this framework, genetic variation at *Irq30* may differentially influence recruitment dynamics and cellular accumulation, leading to context-dependent effects on disease severity across infections.

Beyond these illustrative loci, a broader pattern emerges linking disease severity to composition of the myeloid compartment and expression of MHC II, a marker associated with antigen presenting function. Traits related to macrophages, inflammatory monocytes, and MHC II expression were consistently identified as predictors and mediators of disease severity. In addition to the previously described mediated effects by monocyte-related traits at *HrS43* and *Irq30*, we find evidence for mediation of *HrS48*-driven effects on disease severity by patrolling monocytes, with increased patrolling monocytes having a protective effect on SARS-CoV-2 disease (Supplementary Table 4).

Increased MHC II expression on dendritic cells (DCs), macrophages and monocyte subsets was associated with reduced disease severity in SARS-CoV-infected mice. Specifically, MHC II expression on inflammatory monocytes was identified as predictive of weight loss and as a mediator of *Irq33*-driven disease severity after SARS-CoV infection, having a large protective effect on both weight loss and lung congestion. Notably, *Irq33* also regulates MHC II expression on other macrophage populations (Supplementary Table 3) and maps near *Ciita*, the master regulator of MHC II transcription, making *Ciita* a compelling positional candidate gene underlying this locus (León Machado and Steimle 2021). Additionally, MHC II expression on CD11b^+^ DCs was predictive of weight loss, and on CD103^+^ DCs was a mediator of *Irq18*-driven weight loss, with both traits having a protective effect in SARS-CoV disease (Supplementary Table 2, Supplementary Table 4).

Although we did not detect the same effects of MHC II expression in SARS-CoV-2-infected mice, potentially due to the lower sample size of immune-profiled mice, these results parallel human studies in which decreased expression of HLA-DR, the human analog of MHC II, on monocytes (Giamarellos-Bourboulis et al. 2020; Laing et al. 2020; Schulte-Schrepping et al. 2020; Silvin et al. 2020; Chen et al. 2022) and DCs (Kvedaraite et al. 2021; Hasan et al. 2022; Österberg et al. 2025) is a hallmark of severe COVID-19. These results support a model in which early preservation of MHC II expression, consistent with maintenance of antigen presenting function, promotes effective immune coordination and limits immunopathology, and warrant future studies with larger immune datasets to investigate whether MHC II expression mediates genetic risk in SARS-CoV-2.

A major determinant of immune homeostasis and immune responses to infection is sex, with differences in innate and adaptive immunity contributing to sex-specific susceptibility and outcomes across many viral infections (Klein and Flanagan 2016; Chaulagain et al. 2026). Consistent with these observations, we identified sex-dependent associations between multiple immune traits and disease severity, indicating that the relationship between lung immune composition and coronavirus disease severity differs between males and females. Sex-by-immune trait interaction effects were comparable in magnitude to virus-by-immune trait interaction effects, indicating that sex is an important modifier of immune effects on disease severity. The direction of these interaction effects suggests attenuated immune effects on disease severity in male mice for many, but not all, immune traits. In addition, the overall sex effect indicates reduced disease severity in males across viruses after accounting for immune composition.

In humans, males experience more severe acute SARS and COVID-19 than females (Karlberg 2004; Chaulagain et al. 2026), which is generally inconsistent with our results. However, our mouse population was designed to investigate immunopathology, which is known to contribute to COVID-19 severity in later disease phases, and females generally exhibit stronger immune responses (Klein and Flanagan 2016). This may contribute to immunopathology in some settings—females are at greater risk of long-term symptoms of post-acute sequelae of SARS-CoV-2, and of inflammatory and autoimmune disease more broadly (Klein and Flanagan 2016; Chaulagain et al. 2026). Moreover, the direction of sex effects on immune responses in humans varies with SARS-CoV-2 variant (Chaulagain et al. 2026), highlighting the complexity of sex-dependent immune regulation.

Overall, the findings of our study suggest that conserved host susceptibility loci can influence coronavirus disease severity through immune pathways that are often virus-specific, with MHC II expression and myeloid cell abundance emerging as recurrent themes. By integrating variable selection, genetic mapping, and mediation analysis, this study provides a systems genetics framework for rigorous statistical analysis of the interaction between host genetic variation, immune responses, and disease severity. We find that genetic variation shapes immune mechanisms underlying coronavirus disease outcomes, and highlight immune processes with relevance across mouse models and human coronavirus disease.

## Supplementary Material

iyag209_Supplementary_Data

## Data Availability

The data that support the findings of this study and code to reproduce the results are openly available on GitHub (https://github.com/erisemberg/CoV-immune). Supplemental material available at GENETICS online.

## Appendix A: Immune trait pre-processing

Flow cytometry data were from three panels: lymphoid, myeloid, and granulocyte and rare DC. All myeloid and granulocyte panels were gated by the same analyst, but lymphoid data were gated by two different analysts: experiments done between 6/20/19 and 1/25/20 were gated by one analyst, with those between 6/20/19 and 11/12/19 being gated twice (by the same analyst), and experiments done between 3/6/20 and 4/15/21 were gated by a second analyst. All analysts used the same gating scheme.

We integrated the two datasets for experiments between 06/20/19 and 11/12/19 by correcting for an overall effect of the second batch of measurements and then averaging the two values. Thus, if the second measurements are higher or lower than the first measurements on average, we will not introduce that bias into the averaged trait. The protocol is as follows:

1. For a given trait with two measurements per mouse, let $y_{i,1}$ be the first measurement and $y_{i,2}$ be the second measurement. We fit the following linear model:
  $$y_{i,1}∼\mu +\beta y_{i,2}$$
2. To correct for an overall effect of the second batch of measurements, create a corrected version of the second value as follows:
  $$y_{i,2}^{\mathrm{corrected}}=\hat{\mu }+\hat{\beta }y_{i,2}$$
3. Average the first value with the corrected second value:
  $$y_{i}^{\mathrm{avg}}=\mathrm{mean}(y_{i,1},y_{i,2}^{\mathrm{corrected}})$$

All flow cytometry data were logit-transformed, a common transformation for proportional data that has been shown to eliminate bias in subsequent statistical inference on flow cytometry data (Seiffert et al. 2024). Flow experiment batch effects were handled by fitting the following linear model for all traits,

trait∼sex+infection+(sex×infection)+batch

using the lmer function from the R package lme4 (Bates et al. 2015), where batch is a random effect. Best linear unbiased predictors (BLUPs) for batch effects were removed from the logit-transformed data (using the ranef function) and the resulting data were centered and scaled. In models accounting for both batch effects and gater effects (with batch nested within gater), there were no significant gater effects, so we determined that modeling batch effects was sufficient to account for gater-related variation.

## Appendix B: Bayesian imputation and variable selection

We performed imputation and variable selection simultaneously so that regression estimates and inclusion probabilities reflected imputation uncertainty. In each iteration of the Gibbs sampler, the missing immune data were imputed in the *imputation phase* and then effects were estimated using nonmissing covariates and the current imputed version of the immune data in the *regression phase*.

### Imputation model

Missing immune data were imputed using a Matrix-Normal-Inverse-Wishart (MNIW) model as described by Soch (2020). Let $Z\in R^{n\times q}$ be the matrix containing partially missing immune traits and $X\in R^{n\times p}$ be the fixed-effect design matrix containing fully observed sex and infection covariates. Z follows a matrix normal distribution (an extension of the multivariate normal distribution):

(B1 )
$$Z|B,\Sigma ∼{MN}_{n\times q}(XB,I_{n},\Sigma ),$$

where B is the unknown p×q matrix of regression coefficients, $I_{n}$ is the n×n identity matrix, i.e. the rows are independent, and Σ is the unknown q×q residual covariance matrix. Equivalently, each *q*-length row of Z, $z_{i}^{T}$ for i∈{1,…,n} follows a multivariate normal distribution: $z_{i}^{T}|B,\Sigma ∼N_{q}(x_{i}^{T}B,\Sigma )$.

We use a matrix-normal prior on B with p×q prior mean matrix $M_{0}$, p×p prior row covariance matrix $\Lambda_{0}$, and q×q column covariance matrix Σ (same Σ as above), and an inverse-Wishart prior on Σ:

(B2 )
$$p(B)={MN}_{\;p\times q}(M_{0},\Lambda_{0},\Sigma )$$

(B3 )
$$p(\Sigma )=W^{-1}(\nu_{0},S_{0})$$

with weakly informative prior hyperparameters $M_{0}=0$, $\Lambda_{0}=nI_{p}$, $\nu_{0}=q+2$, and $S_{0}=(\nu_{0}-q-1)I_{q}$. This results in a conjugate matrix-normal posterior on B:

(B4 )
$$B|\Sigma ∼{MN}_{\;p\times q}(M_{n},\Lambda_{n},\Sigma ),$$

equivalently sampled as: $\text{vec}(B)|\Sigma =N_{nq}(\text{vec}(M_{n}),\Sigma ⊗\Lambda_{n})$, where $\Lambda_{n}=(\Lambda_{0}^{-1}+X^{T}X{)}^{-1}$ and $M_{n}=\Lambda_{n}(\Lambda_{0}^{-1}M_{0}+X^{T}Z)$; and the residual covariance matrix Σ is updated by drawing its precision matrix Ω from a Wishart distribution:

(B5 )
$$\Omega ∼W(\nu_{n},S_{n}^{-1})$$

where $\nu_{n}=\nu_{0}+n$ and $S_{n}=S_{0}+Z^{T}Z+M_{0}\Lambda_{0}^{-1}M_{0}-M_{n}^{T}\Lambda_{n}^{-1}M_{n}$, and then inverting to recover $\Sigma =\Omega^{-1}$.

After updating B and Σ, imputation proceeds based on a conditional multivariate normal model as described in Chapter 7.5 of Hoff (2009). For each individual i=1,…,n, let $z_{i}^{T}$ be the *q*-length row vector of immune traits (row *i* of Z). Under the model:

$$z_{i}^{T}|B,\Sigma ∼N_{q}(\mu_{i}^{T},\Sigma )$$

where $\mu_{i}^{T}=x_{i}^{T}B$, we partition the observation vector $z_{i}$, its mean $\mu_{i}$, and the covariance matrix Σ according to observed *a* and missing *b* immune traits:

$$z_{i}=\left(\begin{array}{c} z_{i,a}\\ z_{i,b} \end{array}\right),\;\mu_{i}=\left(\begin{array}{c} \mu_{i,a}\\ \mu_{i,b} \end{array}\right),\;\Sigma =\left(\begin{array}{cc} \Sigma_{aa} & \Sigma_{ab}\\ \Sigma_{ba} & \Sigma_{bb} \end{array}\right).$$

We draw the missing data $z_{i,b}$ from the conditional multivariate normal:

$$z_{i,b}|z_{i,a},B,\Sigma ∼N(\theta_{i,b},\Sigma_{b|a}),$$

where the conditional mean $\theta_{i,b}=\mu_{i,b}+\Sigma_{ba}\Sigma_{aa}^{-1}(z_{i,a}-\mu_{i,a})$ and the conditional covariance $\Sigma_{b|a}=\Sigma_{bb}-\Sigma_{ba}\Sigma_{aa}^{-1}\Sigma_{ab}$.

### Regression model

After imputing missing covariate data, we performed a linear regression with weight loss as a function of covariates modeled as fixed effects and immune trait predictors that are subject to variable selection. Each immune trait contributed four predictors: a main effect, a trait-by-sex effect, a trait-by-SARS-CoV-infection effect, and a trait-by-SARS-CoV-2-infection effect. To identify which of these variables were important in predicting weight loss, we applied Stochastic Search Variable Selection (SSVS) (George and McCulloch 1993, 1997) using a discrete normal mixture prior (Supplementary Fig. 2a). This prior shrinks estimated effects to encourage sparsity and provides posterior inclusion probabilities (PIPs) for each predictor.

Let $y\in R^{n}$ be the fully observed disease severity trait (weight loss as measured by the AAC statistic), $X\in R^{n\times p}$ be the fixed-effect design matrix containing fully observed sex and infection covariates, $Z\in R^{n\times q}$ be the matrix containing imputed immune traits, and $Z_{\mathrm{int}}\in R^{n\times (4\times q)}$ be the matrix containing imputed immune traits and interaction terms with sex and infection, created from the current Z in each iteration. Since all covariates in X are coded as [−0.5,0.5], the resulting $Z_{\mathrm{int}}$ is still centered and scaled. The multivariate normal likelihood for the outcome is:

(B6 )
$$y|\beta ,\delta ,\sigma^{2}∼N_{n}(X\beta +Z\delta ,\sigma^{2}I_{n}),$$

where β is a *p*-length vector of estimated fixed-effect coefficients, δ is a *q*-length vector of estimated coefficients subject to selection, and $\sigma^{2}$ is the residual variance. We apply a multivariate normal prior on the β and an inverse-gamma prior on $\sigma^{2}$:

(B7 )
$$\beta ∼N_{p}(\mu_{\beta 0},I_{p}\phi^{2})$$

(B8 )
$$\sigma^{2}∼IG(\text{shape}=a_{0},\text{rate}=b_{0}).$$

with weakly informative prior hyperparameters $\mu_{\beta 0}=0$, $\phi^{2}=10\times \text{svar}(y)$, $a_{0}=3$, $b_{0}=(a_{0}-1)\times \text{svar}(y)$, where svar(y) is the sample variance of the fully observed y.

SSVS (George and McCulloch 1993, 1997) is used to apply variable selection to the predictors in $Z_{\mathrm{int}}$. This involves a discrete normal mixture (spike-and-slab) prior on the immune effects $\delta =(\delta_{1},\ldots ,\delta_{q})$, with binary inclusion indicators for each predictor $\gamma_{j}\in (0,1)$ and inclusion probability *ψ*:

(B9 )
$$\delta_{j}=(1-\gamma_{j})N(0,\omega_{j}^{2})+\gamma_{j}N(0,\tau_{j}^{2})$$

(B10 )
$$\gamma_{j}|\psi ∼\mathrm{Bernoulli}(\psi )$$

(B11 )
$$\psi ∼\mathrm{Beta}(a_{\psi },b_{\psi })$$

where $\tau^{2}$ is the “spike” variance and $\omega^{2}=\tau^{2}c_{0}$ is the “slab” variance, such that $\omega^{2}\gg \tau^{2}$. Hyperparameters were chosen as $\tau^{2}=2.5\times 10^{-5}$, $c_{0}=300$, with $a_{\psi }=2$ and $b_{\psi }=7$, which centers the prior *ψ* around $0.\bar{2}$. Sensitivity analysis revealed that the choice of $a_{0}$, $\tau^{2},c_{0},a_{\psi }$, and $b_{\psi }$ did not meaningfully affect variable selection results.

The posterior Gibbs updates on β, *ψ*, γ, δ, and $\sigma^{2}$ proceed as follows.

(B12 )
$$\beta ∼N_{p}(A^{-1}B,A^{-1})$$

where $A=X^{T}X/\sigma^{2}+I_{p}\phi^{2}$ and $B=X^{T}(y-Z\delta )/\sigma^{2}+I_{p}\mu_{\beta 0}/\phi^{2}$.

(B13 )
$$\psi ∼\text{Beta}\left(a_{\psi }+\sum \gamma_{j},b_{\psi }+4q-\sum \gamma_{j}\right).$$

Inclusion indicators $\gamma_{j}$ are updated individually using a collapsed Bayes factor posterior odds update (integrating out $\delta_{j}$) (Van Dyk and Park 2008):

1. For each predictor *j*, partial residuals (residuals after removing all terms except the *j*th term) are computed as $r_{j}=y-X\beta -Z\delta +Z_{j}\delta_{j}$, to isolate the evidence for predictor *j* given the current state of the rest of the model.
2. The collapsed Bayes factor (with $\delta_{j}$ integrated out), conditional on current $\delta_{-j}$, is calculated by comparing the marginal likelihoods under spike versus slab priors:
  $$\begin{array}{rl} \log\;BF_{j} & =\frac{\;p(r_{j}|\gamma_{j}=1)}{p(r_{j}|\gamma_{j}=0)}\\ & =\frac{1}{2}[(\log\;s_{\mathrm{slab}}^{2}-\log\;\omega^{2})-(\log\;s_{\mathrm{spike}}^{2}-\log\;\tau^{2})\\ & \;+\frac{m_{\mathrm{slab}}^{2}}{s_{\mathrm{slab}}^{2}}+\frac{m_{\mathrm{spike}}^{2}}{s_{\mathrm{spike}}^{2}}] \end{array}$$
  where $s_{\mathrm{slab}}^{2}=(\|Z_{j}{\|}^{2}/\sigma^{2}+1/\omega^{2}{)}^{-1}$, $m_{\mathrm{slab}}=s_{\mathrm{slab}}^{2}\cdot Z_{j}^{T}r_{j}/\sigma^{2}$, and analogously for the spike prior. The use of $\|Z_{j}{\|}^{2}=\sum_{i}z_{ij}^{2}$ enables efficient computation of conditional variances without repeated matrix inversions.
3. The posterior inclusion probability is then:
  $$P(\gamma_{j}=1|\cdot )={\mathrm{logit}}^{-1}(\log\psi -\log(1-\psi )+\log BF_{j}),$$
4. and the inclusion indicator for predictor *j* is drawn from:
  (B14 ) $$\gamma_{j}∼\mathrm{Bernoulli}(P(\gamma_{j}=1|\cdot ))$$

Given current γ values, immune effects δ are drawn from their conditional normal posterior:

(B15 )
$$\delta |\cdot ∼N(A^{-1}B,A^{-1})$$

where $A=Z_{\mathrm{int}}^{T}Z_{\mathrm{int}}/\sigma^{2}+\text{diag}(D_{\gamma }^{-1})$, $B=Z_{\mathrm{int}}^{T}(y-X\beta )/\sigma^{2}$, and $\{D_{\gamma }{\}}_{\;j,j}=\omega^{2}$ if $\gamma_{j}=1$ and $\{D_{\gamma }{\}}_{\;j,j}=\tau^{2}$ if $\gamma_{j}=0$. Finally, the residual variance $\sigma^{2}$ is updated from its inverse-gamma posterior:

(B16 )
$$\begin{array}{rl} \sigma^{2}|\cdot ∼IG( & \text{shape}=a_{0}+\frac{n}{2},\\ & \text{rate}=b_{0}+\frac{1}{2}(y-X\beta -Z_{\mathrm{int}}\delta {)}^{T}(y-X\beta -Z_{\mathrm{int}}\delta )) \end{array}$$

The Gibbs sampler was run for 5,000 iterations with a burn-in period of 1,000 iterations. To prevent premature convergence to an empty model during early learning, two “warm start” strategies are applied for the first 50 iterations: (i) the inclusion probability *ψ* is fixed at its prior mean $a_{\psi }/(a_{\psi }+b_{\psi })$, and (ii) variables identified as top candidates via ordinary least squares regression are held in the model ($\gamma_{j}=1$).

### Sensitivity analysis

We analyzed the sensitivity of the variable selection model to hyperparameter choice by fitting the model over a grid of hyperparameter values: $a_{\psi }\in (1,2,5)$, $b_{\psi }\in (5,7,10)$, $\tau^{2}\in (1\times 10^{-5},2.5\times 10^{-5},5\times 10^{-5})$, $c_{0}\in (100,300,600)$, and $a_{0}\in (3,5)$ (with $b_{0}$ calculated from $a_{0}$ such that the expectation of the inverse-gamma prior on $\sigma^{2}$, $\frac{b_{0}}{a_{0}-1}$, is equal to svar(y)). Leave-one-out cross-validation indicated no statistically meaningful differences in predictive performance, as measured by the expected log pointwise predictive density, ELPD, calculated using Pareto smoothed importance sampling as implemented in the R package loo (Vehtari et al. 2017), so hyperparameters were chosen for sparsity behavior and interpretability.
