# Supplementary material for: Systems genetics analysis reveals immune mediators of coronavirus disease severity in mice
Source: Genetics. 2026 Aug 21;234(2):iyag209. doi: 10.1093/genetics/iyag209 (PMC13643782; doi:10.1093/genetics/iyag209)
Supplement: Supplementary file 1 [file iyag209_supplementary_data.zip › Supplementary_Figure_3_GENETICS-2026-309484.pdf]

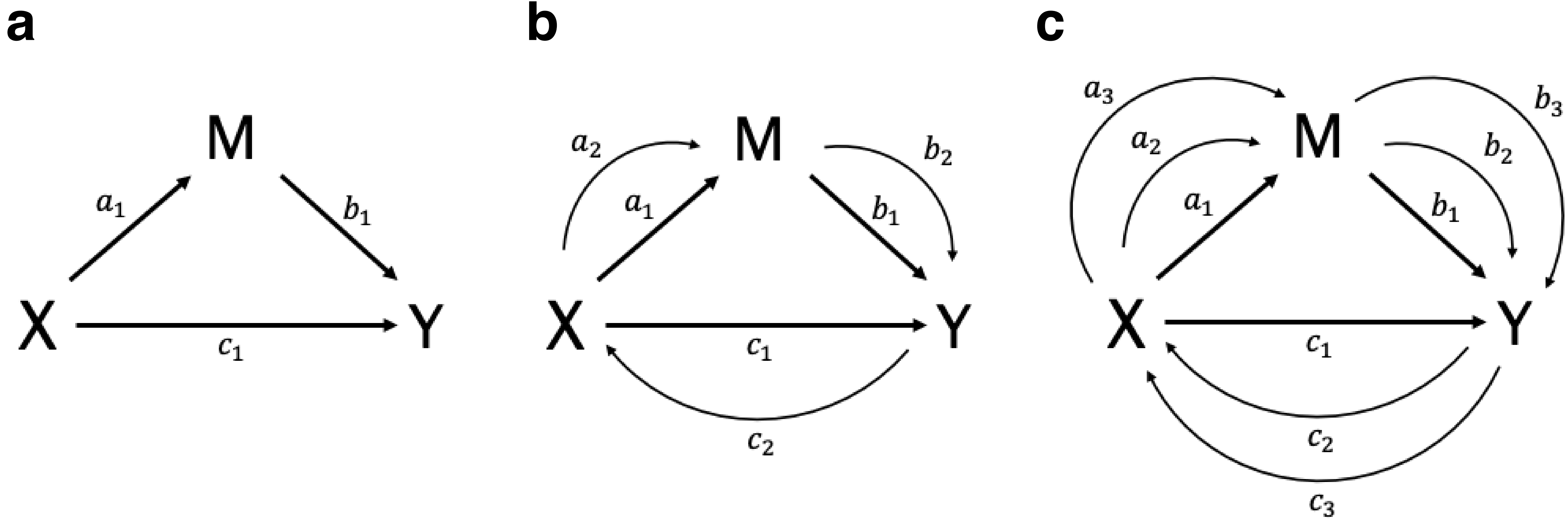
a
M
X
Y
a_1
b_1
c_1
b
M
X
Y
a_1
a_2
b_1
b_2
c_1
c_2
c
M
X
Y
a_1
a_2
a_3
b_1
b_2
b_3
c_1
c_2
c_3
