# Supplementary material for: Systems genetics analysis reveals immune mediators of coronavirus disease severity in mice
Source: Genetics. 2026 Aug 21;234(2):iyag209. doi: 10.1093/genetics/iyag209 (PMC13643782; doi:10.1093/genetics/iyag209)
Supplement: Supplementary file 1 [file iyag209_supplementary_data.zip › Supplementary_Figure_4_GENETICS-2026-309484.pdf]

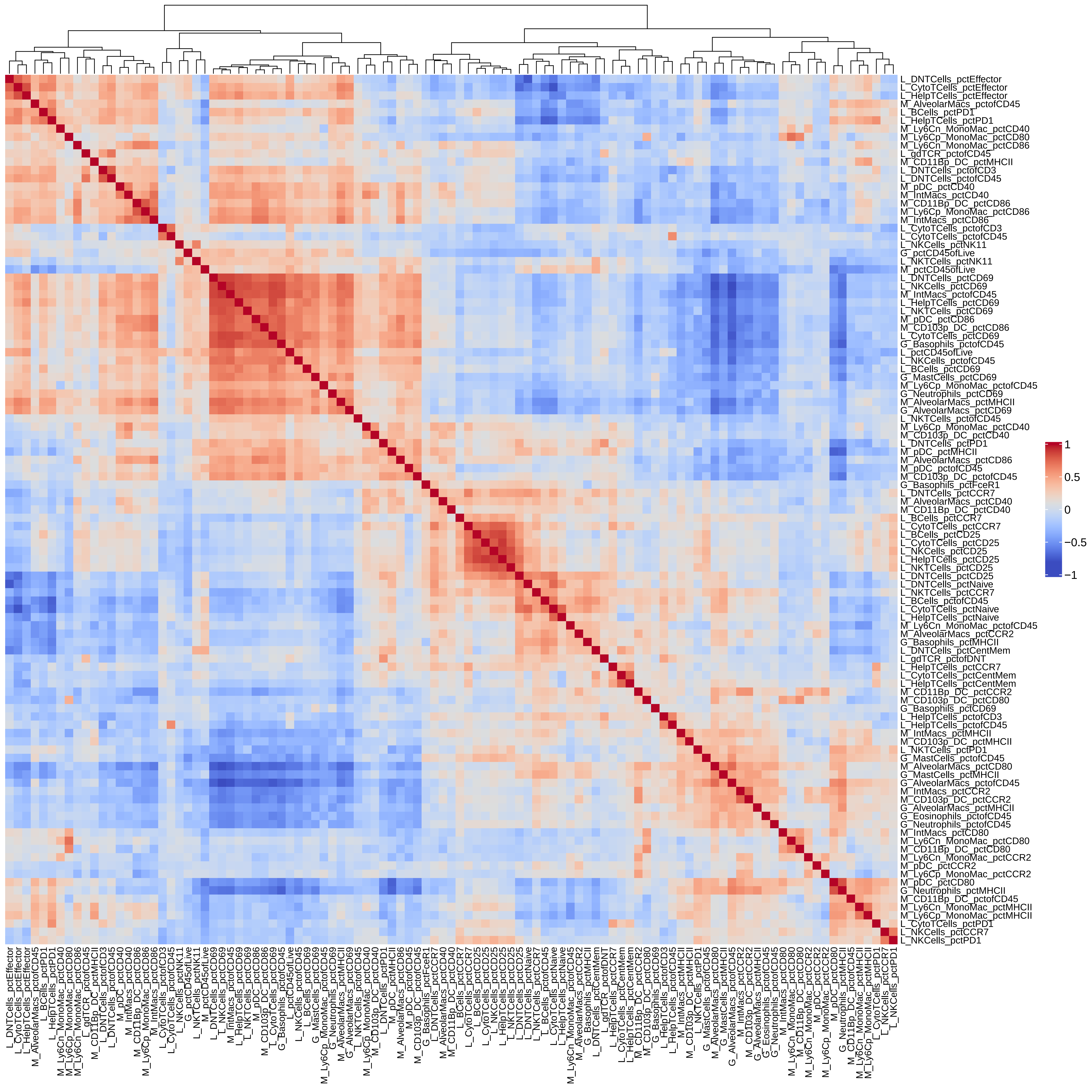
L_DNTCells_pctEffector
L_CytoTCells_pctEffector
L_HelpTCells_pctEffector
M_AlveolarMacs_pctofCD45
L_BCells_pctPD1
L_HelpTCells_pctPD1
M_Ly6Cn_MonoMac_pctCD40
M_Ly6Cp_MonoMac_pctCD80
M_Ly6Cn_MonoMac_pctCD86
L_gdTCR_pctofCD45
M_CD11Bp_DC_pctMHCII
L_DNTCells_pctofCD3
L_DNTCells_pctofCD45
M_pDC_pctCD40
M_IntMacs_pctCD40
M_CD11Bp_DC_pctCD86
M_Ly6Cp_MonoMac_pctCD86
M_IntMacs_pctCD86
L_CytoTCells_pctofCD3
L_CytoTCells_pctofCD45
L_NKCells_pctNK11
G_pctCD45ofLive
L_NKTCells_pctNK11
M_pctCD45ofLive
L_DNTCells_pctCD69
L_NKCells_pctCD69
M_IntMacs_pctofCD45
L_HelpTCells_pctCD69
L_NKTCells_pctCD69
M_pDC_pctCD86
M_CD103p_DC_pctCD86
L_CytoTCells_pctCD69
G_Basophils_pctofCD45
L_pctCD45ofLive
L_NKCells_pctofCD45
L_BCells_pctCD69
G_MastCells_pctCD69
M_Ly6Cp_MonoMac_pctofCD45
G_Neutrophils_pctCD69
M_AlveolarMacs_pctMHCII
G_AlveolarMacs_pctCD69
L_NKTCells_pctofCD45
M_Ly6Cp_MonoMac_pctCD40
M_CD103p_DC_pctCD40
L_DNTCells_pctPD1
M_pDC_pctMHCII
M_AlveolarMacs_pctCD86
M_pDC_pctofCD45
M_CD103p_DC_pctofCD45
G_Basophils_pctFceR1
L_DNTCells_pctCCR7
M_AlveolarMacs_pctCD40
M_CD11Bp_DC_pctCD40
L_BCells_pctCCR7
L_CytoTCells_pctCCR7
L_BCells_pctCD25
L_CytoTCells_pctCD25
L_NKCells_pctCD25
L_HelpTCells_pctCD25
L_NKTCells_pctCD25
L_DNTCells_pctCD25
L_DNTCells_pctNaive
L_NKTCells_pctCCR7
L_BCells_pctofCD45
L_CytoTCells_pctNaive
L_HelpTCells_pctNaive
M_Ly6Cn_MonoMac_pctofCD45
M_AlveolarMacs_pctCCR2
G_Basophils_pctMHCII
L_DNTCells_pctCentMem
L_gdTCR_pctofDNT
L_HelpTCells_pctCCR7
L_CytoTCells_pctCentMem
L_HelpTCells_pctCentMem
M_CD11Bp_DC_pctCCR2
M_CD103p_DC_pctCD80
G_Basophils_pctCD69
L_HelpTCells_pctofCD3
L_HelpTCells_pctofCD45
M_IntMacs_pctMHCII
M_CD103p_DC_pctMHCII
L_NKTCells_pctPD1
G_MastCells_pctofCD45
M_AlveolarMacs_pctCD80
G_MastCells_pctMHCII
G_AlveolarMacs_pctofCD45
M_IntMacs_pctCCR2
M_CD103p_DC_pctCCR2
G_AlveolarMacs_pctMHCII
G_Eosinophils_pctofCD45
G_Neutrophils_pctofCD45
M_IntMacs_pctCD80
M_Ly6Cn_MonoMac_pctCD80
M_CD11Bp_DC_pctCD80
M_Ly6Cn_MonoMac_pctCCR2
M_pDC_pctCCR2
M_Ly6Cp_MonoMac_pctCCR2
M_pDC_pctCD80
G_Neutrophils_pctMHCII
M_CD11Bp_DC_pctofCD45
M_Ly6Cn_MonoMac_pctMHCII
M_Ly6Cp_MonoMac_pctMHCII
L_CytoTCells_pctPD1
L_NKCells_pctCCR7
L_NKCells_pctPD1
1
0.5
0
−0.5
−1
