# Supplementary material for: Systems genetics analysis reveals immune mediators of coronavirus disease severity in mice
Source: Genetics. 2026 Aug 21;234(2):iyag209. doi: 10.1093/genetics/iyag209 (PMC13643782; doi:10.1093/genetics/iyag209)
Supplement: Supplementary file 1 [file iyag209_supplementary_data.zip › Supplementary_Figure_Legends_GENETICS-2026-309484.docx]

**Supplementary Fig. 1. Supplemental trait information**. a) Illustration of “area above the curve” (AAC) statistic used to summarize weight loss trajectories. b) Association between viral titer and weight loss in virus-infected mice, with $P$-values determined by linear regression. c) Association between viral titer and lung congestion score in virus-infected mice, with $P$-values determined by proportional odds regression. d) Counts of immune traits by category. e) Variance partitioning analysis on immune traits (see Materials and Methods).

**Supplementary Fig. 2. Bayesian variable selection model performance**. a) Discrete normal mixture (“spike-and-slab”) prior defined by chosen hyperparameters $\tau^{2}=2.5\times{10}^{-5}$ and $c_{0}=300$ (see Materials and Methods and Appendix B). b) Empirical cumulative distribution function (ECDF) of rank-based Gelman-Rubin statistic ($\hat{R}$) computed across all monitored parameters from multiple independent Gibbs sampling chains. The ECDF at $\hat{R}$ threshold $x$ represents the fraction of parameters with $\hat{R}\leq x$. $\hat{R}$ values concentrated near 1 (i.e., $\hat{R}\lesssim1.01-1.05$) indicate good agreement across chains and are consistent with adequate mixing and convergence. c) Histogram of in-sample Bayesian $R^{2}$ values (Gelman et al., 2019) calculated from all post-burn in samples across all chains. d) Histogram of out-of-sample Bayesian $R^{2}$ values calculated across 5 repeats of 10-fold cross-validation (CV). e) Mean posterior inclusion probability (PIP) across CV folds versus selection stability at our chosen PIP threshold pf 0.23. f) Histogram of pairwise Jaccard similarity of selected sets of variables across CV folds.

**Supplementary Fig. 3. Directed acyclical graphs (DAGs) representing group-specific mediation analysis**. DAGs show edges connecting exposure ($X$), mediator ($M$) and outcome ($Y$) in single-group, two-group, and three-group mediation analysis. In our analyses, $X$ represents a QTL genotype, $M$ is an immune trait, and $Y$ is disease severity (i.e., weight loss or lung congestion). a) Edge $a$ represents the $X\to M$ effect, edge $b$ represents the $M\to Y$ effect, and $a$ and $b$ together capture the mediated or “indirect” effect. Edge $c$ represents the “direct” $X\to Y$ effect. b) Edges $a_{2},b_{2}$, and $c_{2}$ represent the corresponding effects in a second group. c) Edges $a_{3},b_{3}$, and $c_{3}$ represent the corresponding effects in a third group.

**Supplementary Fig. 4. Labeled heatmap of immune trait correlation**. Labeled version of heatmap shown in Fig. 4A. Hierarchical clustering is based on Euclidian distance of the correlation matrix.

**Bibliography**

Gelman A, Goodrich B, Gabry J, Vehtari A. 2019. R-squared for Bayesian Regression Models. The American Statistician. 73(3):307–309. doi:10.1080/00031305.2018.1549100.
